# Crystal structure and Hirshfeld surface analysis of poly[[bis­[μ_4_-*N*,*N*′-(1,3,5-oxadiazinane-3,5-di­yl)bis(carbamoyl­methano­ato)]nickel(II)tetra­potassium] 4.8-hydrate]

**DOI:** 10.1107/S205698902100205X

**Published:** 2021-02-26

**Authors:** Maksym O. Plutenko, Matti Haukka, Alina O. Husak, Turganbay S. Iskenderov, Nurullo U. Mulloev

**Affiliations:** a Department of Chemistry, National Taras Shevchenko University, Volodymyrska Street 64, 01601 Kyiv, Ukraine; b Department of Chemistry, University of Jyvaskyla, P.O. Box 35, FI-40014 Jyvaskyla, Finland; c PBMR Labs Ukraine, Murmanska 1, 02094 Kiev, Ukraine; d The Faculty of Physics, Tajik National University, Rudaki Avenue 17, 734025 Dushanbe, Tajikistan

**Keywords:** crystal structure, nickel(II) complex, template reaction, pseudomacrocyclic ligand, hydrazide-based ligand, SHAPE analysis, Hirshfeld surface analysis

## Abstract

The title compound, {[K_4_Ni_2_(C_7_H_6_N_4_O_7_)_2_]·4.8H_2_O}_*n*_, was obtained as a result of a template reaction between oxalohydrazide­hydroxamic acid, formaldehyde and nickel(II) nitrate followed by partial hydrolysis of the formed inter­mediate. The two independent [Ni(C_7_H_6_N_4_O_7_)]^2–^ complex anions exhibit pseudo-*C*_S_ symmetry and consist of an almost planar metal-containing fragment and a 1,3,5-oxadiazinane ring with a chair conformation disposed nearly perpendicularly with respect to the former. The central Ni^II^ atom has a square-planar N_2_O_2_ coordination arrangement formed by two amide N and two carboxyl­ate O atoms. In the crystal, the nickel(II) complex anions form layers parallel to the *ab* plane. Neighboring complex anion layers are connected by layers of potassium cations for which two of the four independent cations are disordered over two sites [ratios of 0.54 (3):0.46 (3) and 0.9643 (15):0.0357 (15)]. The framework is stabilized by an extensive system of hydrogen bonds where the water mol­ecules act as donors and the carb­oxy­lic O atoms, the amide O atoms and the oxadiazinane N atoms act as acceptors.

## Abstract 2

The complex nickel(II) anion comprises a pseudomacrocyclic hydrazide-based ligand with an L shape. In the crystal, such anions are connected with the potassium cations and the water solvent mol­ecules, forming a three-dimensional polymeric framework, which is stabilized by an extensive system of hydrogen bonds.

## Chemical context   

Coordination compounds of paramagnetic metal ions based on polydentate ligands comprising amide, hydrazide and hydroxamate functional groups are of great inter­est as they often form novel oligonuclear structures with inter­esting supra­molecular features (Mezei *et al.*, 2007 ▸; Strotmeyer *et al.*, 2003 ▸). Frequently, these compounds exhibit unusual magnetic properties (Pavlishchuk *et al.*, 2010 ▸; Gumienna-Kontecka *et al.*, 2007 ▸; Pavlishchuk *et al.*, 2011 ▸; Huang *et al.*, 2014 ▸) and have potential biological activity (Raja *et al.*, 2012 ▸). The use of hydrazide metal complexes as synthons for template reactions has allowed coordination compounds with more complicated, sometimes unpredictable mol­ecular structures to be obtained (Clark *et al.*, 1976 ▸). In particular, for ring-closure reactions, aldehydes (especially formaldehyde) can be used successfully as capping reagents for template condensation, as has been shown in several studies (Fritsky *et al.*, 1998 ▸, 2006 ▸; Tomyn *et al.*, 2017 ▸). Importantly, depending on the nature and coordination preference of the metal ion, the products of the ring-closure reactions can be both macrocyclic or pseudomacrocyclic (Ni^2+^, Cu^2+^; Fritsky *et al.*, 1998 ▸, 2006 ▸; Tomyn *et al.*, 2017 ▸) and macrobicylic (Fe^4+^; Tomyn *et al.*, 2017 ▸, Shylin *et al.*, 2019*a* ▸,*b* ▸).
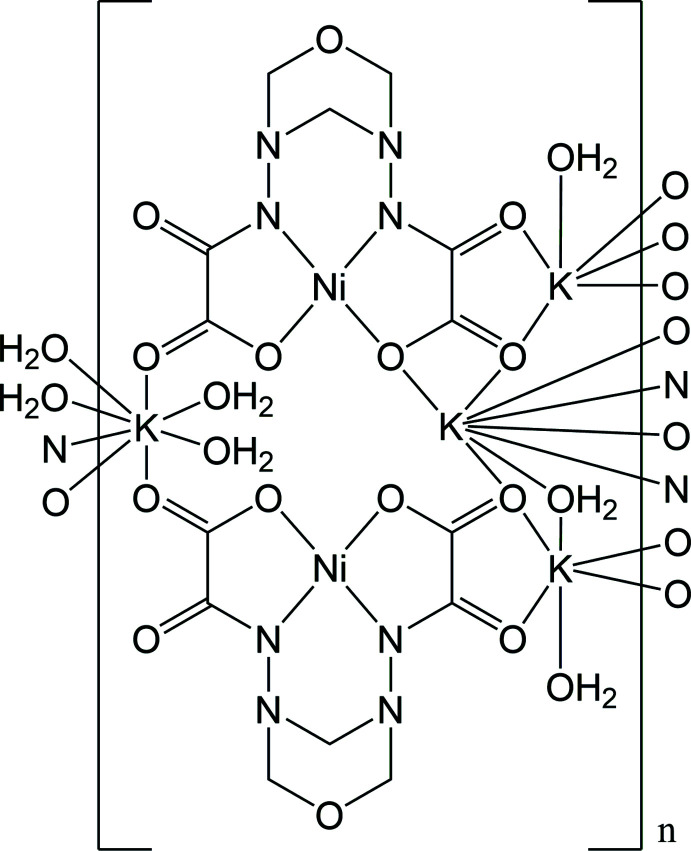


**Figure 1:**
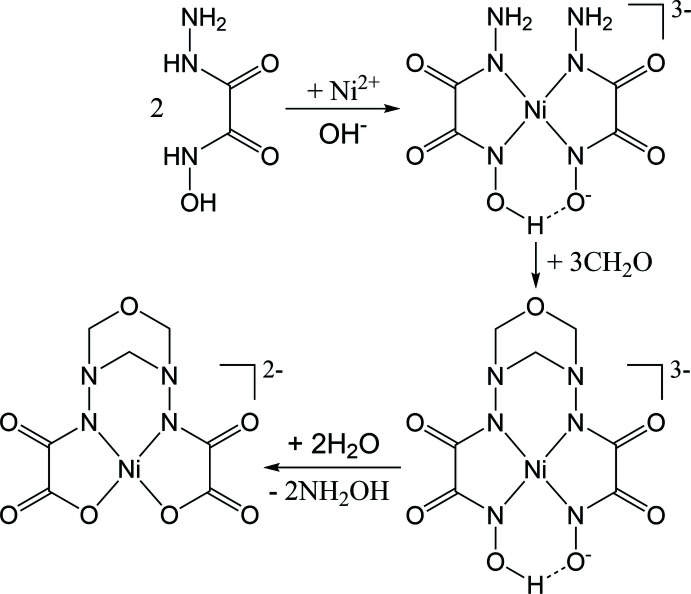
The plausible mechanism for the formation of the [Ni(*L*-2H)]^2–^ anion.

Here, we report the crystal structure of the polymeric title compound {K_4_[Ni(*L*-2H)]_2_·4.8H_2_O}_*n*_ [*L* = *N*,*N*′-(1,3,5-oxadiazinane-3,5-di­yl)bis­(carbamoyl­methanoic acid)] (**1**) obtained as a result of a template reaction between oxalohydrazide­hydroxamic acid, formaldehyde and nickel(II) nitrate followed by partial hydrolysis of the formed inter­mediate. The plausible mechanism of formation for (**1**) includes the deprotonation of oxalohydrazide­hydroxamic acid and coord­ination to the metal ions in a tetra­dentate mode, followed by template condensation of two hydrazide moieties with three mol­ecules of formaldehyde and metal-promoted hydrolysis of the hydroxamate group of the formed inter­mediate, which eventually results in the formation of the nickel(II) complex anion [Ni(*L*-2H)]^2−^ (Fig. 1 ▸). The crystallization process causes the bonding of such anions with the potassium counter-cations and the water solvent mol­ecules, forming the three-dimensional coordination polymer {K_4_[Ni(*L*-2H)]_2_·4.8H_2_O}_*n*_ (**1**).

## Structural commentary   

**Figure 2:**
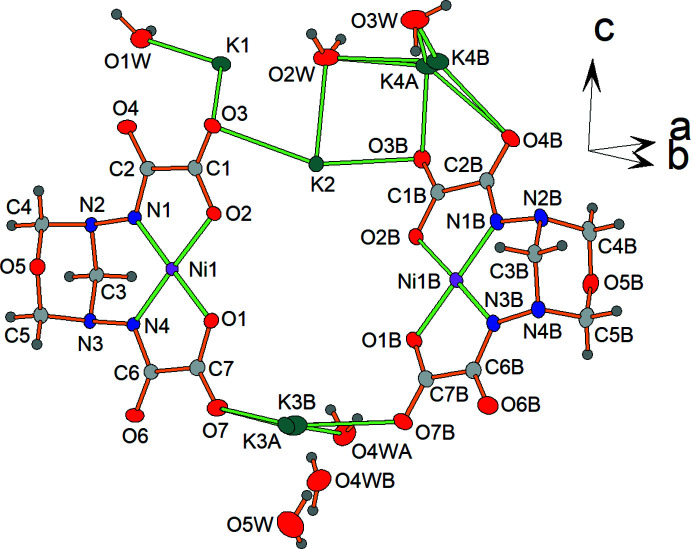
The asymmetric unit of (**1**) with displacement ellipsoids shown at the 50% probability level. The potassium cations K3 and K4 and the solvate water mol­ecule O4*W* are disordered over two positions, namely K3*A* and K3*B*, K4*A* and K4*B*, O4*WA* and O4*WB*, respectively.

The asymmetric unit of (**1**) (Fig. 2 ▸) contains two complex anions [Ni(*L*-2H)]^2–^ (which contain Ni1 and Ni1*B*, respectively), four potassium cations (two of which, K3 and K4, are disordered over two sites) and five solvent water mol­ecules, one of which is disordered over two sets of sites (O4*WA* and O4*WB* in a ratio of 0.8:0.2), and one (O5*W*) that has an occupancy of 0.8.

Both complex anions [Ni(C_7_H_6_N_4_O_7_)]^2–^ have a pseudo-*C*_S_ symmetry with similar bond lengths and angles. Each anion consists of an almost planar metal-containing {NiN_2_O_2_} fragment [the maximum deviation of the atoms involved in the anion from the least-squares plane is 0.1232 (12) Å for the anion centred by Ni1 and −0.1510 (13) Å for the anion centred by Ni1*B*] and an 1,3,5-oxadizdinane ring disposed nearly perpendicularly with respect to the former. The 1,3,5-oxadizdinane ring in each anion adopts a chair conformation. The dihedral angle between the mean planes formed by the non-hydrogen atoms of these two fragments is 87.22 (5)° for the Ni1 anion and 86.89 (5)° for the Ni1*B* anion. Thus, the complex anions reveal an L-like shape.

The ligand mol­ecule (*L*-2H) coordinates in a tetra­dentate {O_carbox­yl_,N_amide_,N_amide_,O_carbox­yl_} mode, thus forming three fused chelate rings (two five-membered and one six-membered). The central Ni^II^ atom of the complex anion has a square-planar coordination arrangement with an N_2_O_2_ chromophore. The deviation of the Ni^II^ atom from the mean plane defined by the four donor atoms is 0.0098 (8) and 0.0116 (9) Å for Ni1 and Ni1*B*, respectively. The Ni—N and Ni—O bond lengths (Table 1 ▸) are in the range 1.8429 (15)–1.8479 (15) and 1.8830 (13)–1.9012 (13) Å, respectively, typical for square-planar nickel(II) complexes with similar tetra­dentate ligands (Fritsky *et al.*, 2004 ▸; Sliva *et al.*, 1997*a* ▸,*b* ▸; Duda *et al.*, 1997 ▸). The bite angles O1—Ni1—N4, N1—Ni1—O2 and N1—Ni1—N4 for both anions (Table 1 ▸) deviate from the ideal value of 90°, conditioned by the formation of five-membered chelate rings. N—N′, N—C and C—O and C=O bond lengths within the (*L*-2H) ligand indicate values typical for the coordinating deprotonated hydrazide and carboxyl groups.

## Supra­molecular features   

**Figure 3:**
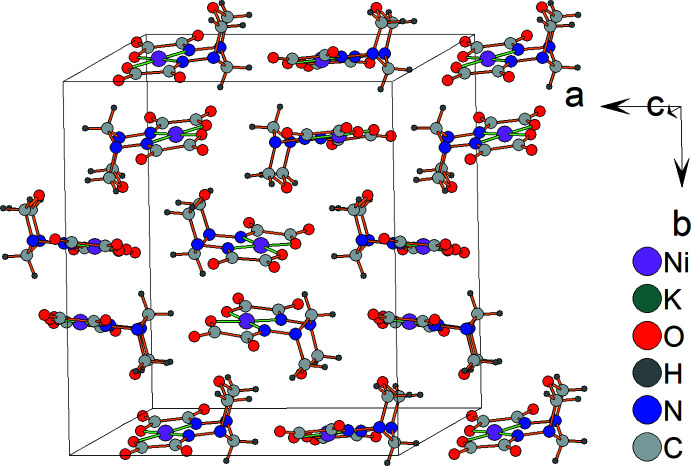
Layers formed by the anionic nickel(II) complexes.

**Figure 4:**
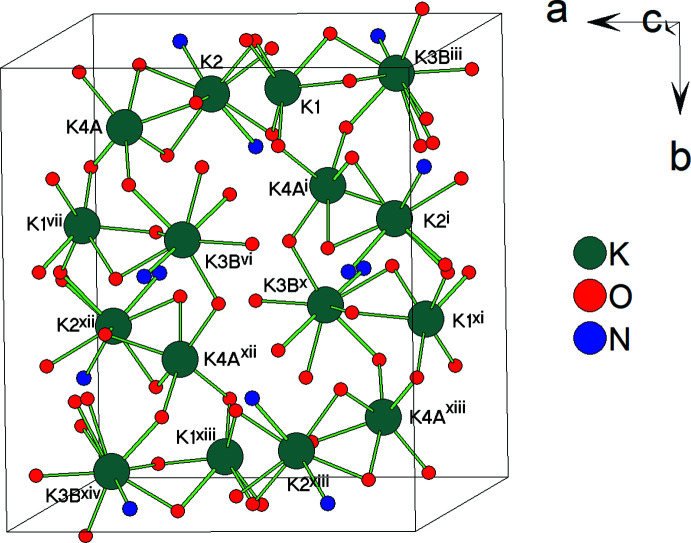
The layer of potassium cations and their coordination arrangement. The minor disordered components of K3 and K4 atoms (K3*A* and K4*B*) are omitted for clarity. [Symmetry codes: (i) *x* − 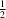, −*y* + 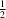, −*z* + 1; (iii) *x* − 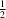, *y*, −*z* + 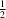; (vi) *x*, −*y* + 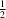, *z* + 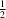; (vii) *x* + 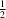, −*y* + 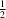, −*z* + 1; (*x*) −*x* + 1, *y* + 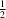, −*z* + 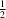; (xi) −*x* + 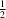, *y* + 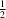, −*z*; (xii) −*x* + 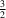, *y* + 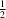, *z*; (xiii) *x* + 1, −*y* + 1, −*z* + 1; (xiv) −*x* + 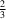, −*y* + 1, *z* + 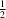].

**Figure 5:**
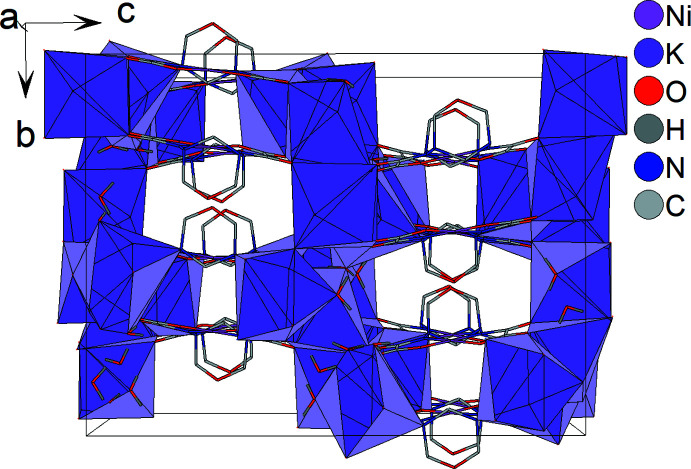
Crystal packing of the title compound in a stick model, showing the coordination polyhedra of the potassium cations in lilac. H atoms of the C—H groups and minor disordered components (K3*A* and K4*B*, O4*WB* water mol­ecule) are omitted for clarity.

In the crystal, the nickel(II) complex anions [Ni(*L*-2H)]^2−^ form layers parallel to *ab* plane (Fig. 3 ▸). Neighboring complex anion layers are sandwiched by layers of potassium counter-cations (Fig. 4 ▸). Thus, complex anion layers and potassium layers are stacked along the *c-*axis direction (Fig. 5 ▸).

The potassium cations are bound to the nickel(II) complex anions through the amide and the carb­oxy­lic O atoms (K1, K4*A*) or through the amide O and the oxadiazinane N atoms (K2, K3*B*). In addition, the potassium cations have contacts with the O atoms of the water mol­ecules, with the amide and the carb­oxy­lic O atoms, and with the oxadiazinane N atoms of neighboring complex anions. For definition of the coordination spheres around the cations, K—O and K—N contacts that do not exceed the sum of the ionic radii by more than 0.2 Å were defined as bonding contacts [the values of the ionic radii were taken from Shannon (1976 ▸)]. The K1, K2 and K4*A* cations exhibit O_6_, O_6_N_2_ and O_6_ coordination sets. As a result of the disorder of the water mol­ecules, the K3*B* site has an O_6_N or O_7_N coordination set. In addition, there are K1⋯O2*W*, K1⋯O7*B* and K2⋯O2*B* remote non-bonding contacts, which are significantly greater than the sum of the ionic radii.

**Figure 6:**
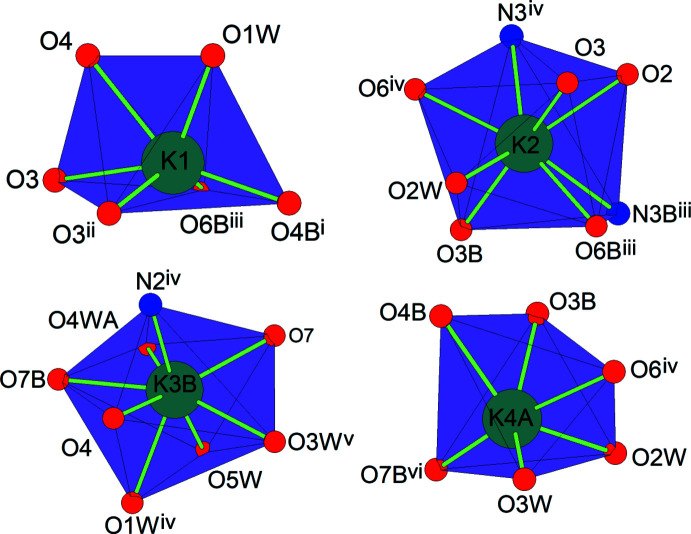
Polyhedral views of the coordination environments for the potassium cations; the minor disordered components of atoms K3 and K4 (K3*A* and K4*B*) are omitted for clarity. [Symmetry codes: (i) *x* − 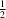, −*y* + 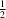, −*z* + 1; (ii) −*x* + 1, −*y*, −*z* + 1; (iii) *x* − 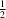, *y*, −*z* + 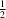; (iv) *x* + 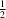, *y*, −*z* + 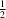; (v) −*x* + 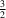, −*y*, *z* − 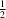; (vi) *x*, −*y* + 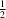, *z* + 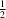].

For an evaluation of the coordination geometry of each potassium cation, the *SHAPE 2.1* software (Llunell *et al.*, 2013 ▸) was used. A SHAPE analysis of the potassium coordination sphere (Table 2 ▸, Fig. 6 ▸) yields the lowest continuous shape measure (CShM) value for a distorted trigonal prism (4.697 for K1), a distorted triangular dodeca­hedron (4.992 for K2), a distorted hexa­gonal bipyramid (13.393 for K3*B*) and a distorted octa­hedron (4.985 for K4*A*). For K2 and K3*B*, comparable CShM values were obtained for a biaugmented trigonal prism (5.698) and an elongated trigonal bipyramid (13.966), respectively.

The polyhedra around neighboring potassium cations are connected with each other through common vertices (K1 with K2, K1 with K4*A*, K3*B* with K4*A*), edges (K1 with K1, K1 with K2, K1 with K3*B*) and faces (K2 with K4*A*). The K—O and K—N bond lengths are normal for potassium cations and close to those reported in the structures of related carboxyl­ate and amide complexes (Fritsky *et al.*, 1998 ▸; Świątek-Kozłowska *et al.*, 2000 ▸; Mokhir *et al.*, 2002 ▸).

**Figure 7:**
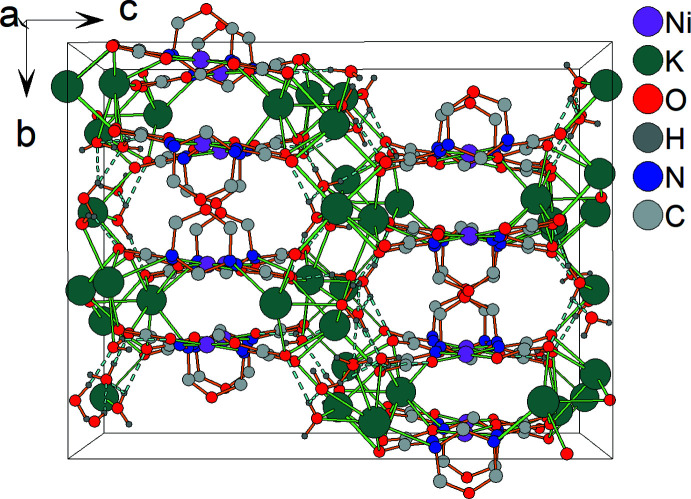
Crystal packing of the title compound. Hydrogen bonds are indicated by dashed lines. H atoms of the C—H groups and minor disordered components (K3*A* and K4*B*, O4*WB* water mol­ecule) are omitted for clarity.

The polymeric framework is stabilized by an extensive system of hydrogen-bonding inter­actions where the water mol­ecules act as donors and the carb­oxy­lic O atoms, the amide O atoms and the oxadizdinane N atoms act as acceptors (Table 3 ▸, Fig. 7 ▸).

## Hirshfeld analysis   

**Figure 8:**
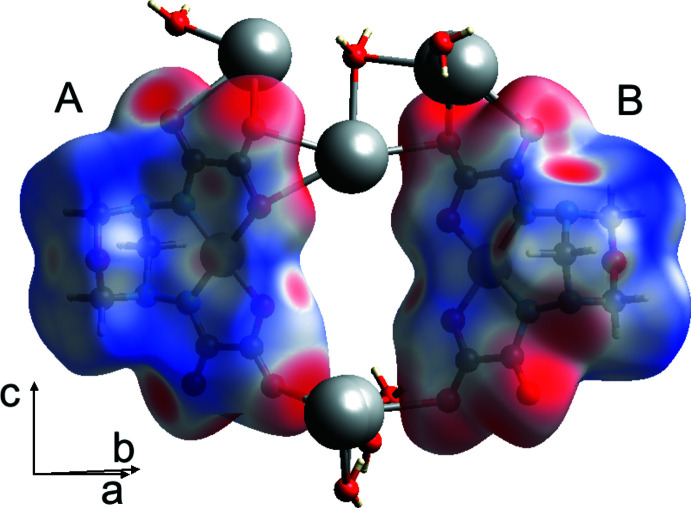
The Hirshfeld surfaces of the two complex anions (A = Ni and B = Ni*B*) mapped over *d*_norm_.

The Hirshfeld surface analysis (Spackman & Jayatilaka, 2009 ▸) was performed and the associated two-dimensional fingerprint plots (McKinnon *et al.*, 2007 ▸) were obtained with *CrystalExplorer17* (Turner *et al.*, 2017 ▸). The Hirshfeld surfaces of the complex anions are colour-mapped with the normalized contact distance (*d*_norm_) from red (distances shorter than the sum of the van der Waals radii) through white to blue (distances longer than the sum of the van der Waals radii). The Hirshfeld surface mapped over *d*_norm_, in the colour range −0.6411 to 0.9651 a.u. for the anion centred by Ni1 (A) and −0,6382 to 0.9607 a.u. for the anion centred by Ni1*B* (B) is shown in Fig. 8 ▸. Both complex anions are connected to the other moieties of the crystal structure mainly through the amide and the carb­oxy­lic O atoms.

**Figure 9:**
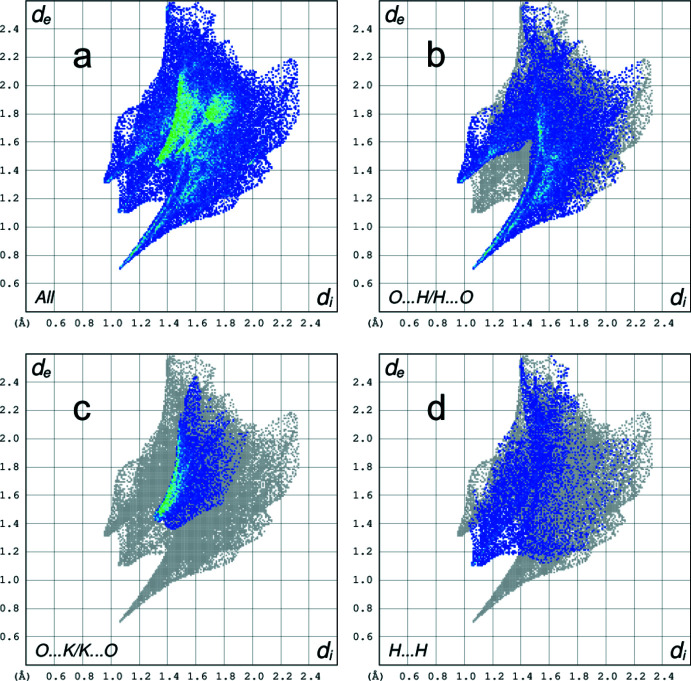
(*a*) Full two-dimensional fingerprint plot of the A complex anion (Ni1), and delineated into (*b*) O⋯H/H⋯O (41.3%) (*c*) O⋯K/K⋯O (15.8%) and (*d*) H⋯H (13.7%) contacts.

A two-dimensional fingerprint plot contains information related to specific inter­molecular inter­actions. The blue colour refers to the frequency of occurrence of the (*d*_i_, *d*_e_) pair with the full fingerprint plot outlined in gray. Figs. 9 ▸ *a* and 10*a* ▸ show the two-dimensional fingerprint plots for the anion centred by Ni1 (A) and by Ni1*B* (B), represented by the sum of the contacts contributing to the Hirshfeld surface in normal mode. The most significant contribution to the Hirshfeld surface is from O⋯H/H⋯O contacts (41.3% for complex *A* and 41.0% for complex *B*, respectively; Fig. 9 ▸ *b* and 10*b*). In addition, O⋯K/K⋯O (15.8% for complex anions *A* and *B*; Fig. 9 ▸ *c* and 10*c*) and H⋯H (13.7% for complex anion *A* and 15.1% for complex anion *B*; Fig. 9 ▸ *d* and 10*d*) are other significant contributions to the total Hirshfeld surface.

## Database survey   

A search of the Cambridge Structural Database (CSD version 5.41, update of November 2019; Groom *et al.*, 2016 ▸) for complexes obtained by hydrazide, aldehyde and 3*d-*metal salt inter­actions gave eleven hits for structures with full atomic coordinates. All these compounds include macrocyclic or pseudo-macrocyclic ligands formed by template binding of several hydrazide groups by aldehyde mol­ecules. The 3*d*-metal ions of these complexes are often in high oxidation states: Cu^III^ (Oliver *et al.*, 1982 ▸; Fritsky *et al.*, 1998 ▸, 2006 ▸) and Fe^IV^ (Tomyn *et al.*, 2017 ▸) complexes have been described.

## Synthesis and crystallization   

A solution of Ni(NO_3_)_2_·6H_2_O (0.073 g, 0.25 mmol) in 5 ml of water was added to a warm solution of oxalohydrazide­hydroxamic acid (0.06 g, 0.5 mmol) in 5 ml of water. The resulting light-green mixture was stirred with heating (320–330 K) for 20 min, and then 1 ml of a 4*M* KOH solution was added. As a result, the color of the solution changed to pink. After 5 min of stirring, 0.03 g of paraformaldehyde (1 mmol) were added, followed by stirring with heating (320–330 K) for 30 min. The resulting orange solution was left for crystallization by slow diffusion of methanol vapor. After two months, orange crystals suitable for X-ray diffraction studies were obtained. The crystals were filtered off, washed with diethyl ether and dried in air.

## Refinement   

Crystal data, data collection and structure refinement details are summarized in Table 4 ▸. The potassium cations K3 and K4 were found to be disordered over two positions with occupancy factors for the major disorder component of 0.54 (3) (K3*B*) and 0.9643 (15) (K4*A*). The solvate water mol­ecule O4*W* appeared to be disordered over two positions with relative occupancies of 0.805 (4) (O4*WA*) and 0.195 (4) (O4*WB*). The solvate water mol­ecule O5*W* was found to be incompatible with the second positions of the water mol­ecule O4*W* and thus was refined with the same occupancy factor as the major fraction of O4*W* as they are linked by a hydrogen bond. The O—H hydrogen atoms were located from a difference-Fourier map and constrained to ride on their parent atoms with *U*_iso_(H) = 1.5*U*_eq_(O). The methyl­ene C—H hydrogen atoms were positioned geometrically and were constrained to ride on their parent atoms, with C—H = 0.99 Å, and *U*_iso_(H) = 1.2*U*_eq_(C).

## Supplementary Material

Crystal structure: contains datablock(s) I. DOI: 10.1107/S205698902100205X/wm5595sup1.cif

Structure factors: contains datablock(s) I. DOI: 10.1107/S205698902100205X/wm5595Isup3.hkl

Supporting information file. DOI: 10.1107/S205698902100205X/wm5595Isup4.cdx

CCDC reference: 2064313

Additional supporting information:  crystallographic information; 3D view; checkCIF report

## Figures and Tables

**Figure 10:**
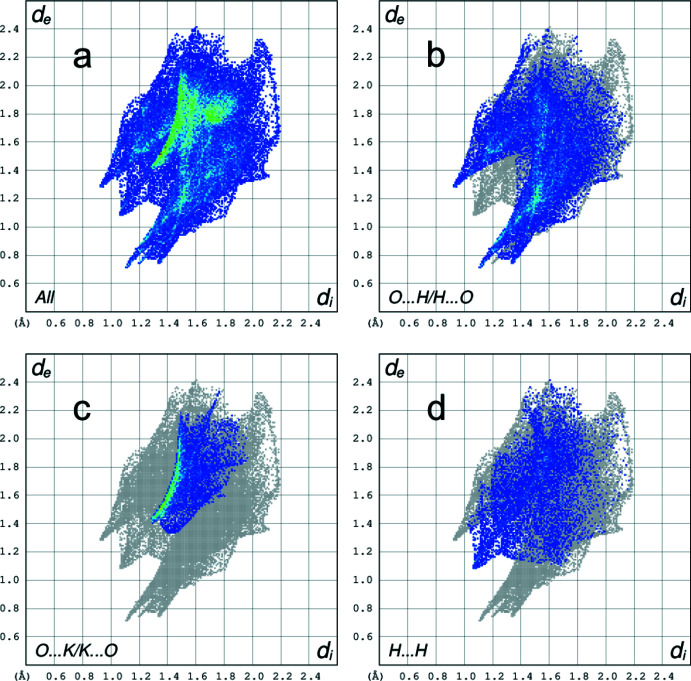
(*a*) Full two-dimensional fingerprint plot of the B complex anion (Ni1*B*), and delineated into (*b*) O⋯H/H⋯O (41.0%) (*c*) O⋯K/K⋯O (15.8%) and (*d*) H⋯H (15.1%) contacts.

**Table 1:** Selected geometric parameters (Å, °)

Ni1—N4	1.8429 (15)	Ni1*B*—N1*B*	1.8436 (16)
Ni1—N1	1.8479 (15)	Ni1*B*—N4*B*	1.8463 (16)
Ni1—O1	1.8830 (13)	Ni1*B*—O2*B*	1.8922 (14)
Ni1—O2	1.9012 (13)	Ni1*B*—O1*B*	1.8975 (14)
			
N4—Ni1—N1	96.01 (7)	N1*B*—Ni1*B*—N4*B*	95.98 (7)
N4—Ni1—O1	85.25 (6)	N1*B*—Ni1*B*—O2*B*	85.01 (6)
N1—Ni1—O1	178.74 (6)	N4*B*—Ni1*B*—O2*B*	177.89 (7)
N4—Ni1—O2	178.28 (7)	N1*B*—Ni1*B*—O1*B*	178.86 (7)
N1—Ni1—O2	85.10 (6)	N4*B*—Ni1*B*—O1*B*	85.08 (7)
O1—Ni1—O2	93.65 (6)	O2*B*—Ni1*B*—O1*B*	93.94 (6)

**Table 2:** Values for continuous shapes measures (CShM) of the polyhedra centred by the potassium cations (only major components for the disordered parts are considered)

Shape	CShM	
	K1	K4*A*
Hexagon (*D*_6*h*_)	30.965	33.688
Penta­gonal pyramid (*C*_5*v*_)	9.924	22.357
Octa­hedron (*Oh*)	13.859	4.985
Trigonal prism (*D*_3*h*_)	4.697	10.581
Johnson penta­gonal pyramid J2 (*C*_5*v*_)	13.919	26.100
	K2	K3*B*
Octa­gon (*D*_8*h*_)	32.591	28.712
Heptagonal pyramid (*C*_7*v*_)	18.314	20.510
Hexagonal bipyramid (*D*_6*h*_)	14.891	13.393
Cube (*Oh*)	14.913	14.525
Square anti­prism (*D*_4*d*_)	6.805	19.105
Triangular dodeca­hedron (*D*_2*d*_)	4.992	17.608
Johnson gyrobifastigium J26 (*D*_2*d*_)	10.479	16.378
Johnson elongated triangular bipyramid J14 (*D*_3*h*_)	23.441	18.219
Biaugmented trigonal prism J50 (*C*_2*v*_)	6.800	16.341
Biaugmented trigonal prism (*C*_2*v*_)	5.698	16.739
Snub diphenoid J84 (*D*_2*d*_)	6.894	15.895
Triakis tetra­hedron (*Td*)	15.016	14.550
Elongated trigonal bipyramid (*D*_3*h*_)	17.893	13.966

**Table 3:** Hydrogen-bond geometry (Å, °)

*D*—H⋯*A*	*D*—H	H⋯*A*	*D*⋯*A*	*D*—H⋯*A*
O1*W*—H1*W*1⋯O6^i^	0.92	1.94	2.840 (2)	166
O1*W*—H2*W*1⋯O5*W* ^ii^	0.83	2.61	3.120 (4)	121
O1*W*—H2*W*1⋯O3*B* ^iii^	0.83	2.23	3.001 (2)	154
O2*W*—H2*W*2⋯O4^iv^	0.93	1.83	2.754 (2)	171
O4*WA*—H2*W*4⋯O4*B* ^ii^	0.91	2.44	2.993 (2)	119
O4*WA*—H2*W*4⋯N2*B* ^ii^	0.91	2.02	2.895 (3)	161
O5*W*—H5*WC*⋯O6*B* ^v^	0.85	1.95	2.774 (3)	162
O4*WB*—H3*W*4⋯O4*B* ^ii^	0.85	2.09	2.848 (9)	149
O4*WB*—H4*W*4⋯O6*B* ^v^	0.88	2.15	3.024 (9)	174

Symmetry codes: (i) -x+{\script{1\over 2}}, -y, z+{\script{1\over 2}}; (ii) x-{\script{1\over 2}}, y, -z+{\script{1\over 2}}; (iii) x-{\script{1\over 2}}, -y+{\script{1\over 2}}, -z+1; (iv) -x+1, -y, -z+1; (v) x-{\script{1\over 2}}, -y+{\script{1\over 2}}, -z.

**Table 4:** Experimental details

Crystal data	
Chemical formula	[K_4_Ni_2_(C_7_H_6_N_4_O_7_)_2_]·4.8H_2_O
*M*_r_	876.66
Crystal system, space group	Orthorhombic, *P* *b* *c* *a*
Temperature (K)	100
*a*, *b*, *c* (Å)	15.0694 (3), 16.9659 (3), 22.1920 (4)
*V* (Å^3^)	5673.74 (18)
*Z*	8
Radiation type	Mo *K*α
μ (mm^−1^)	2.01
Crystal size (mm)	0.31 × 0.26 × 0.23

Data collection	
Diffractometer	Bruker Kappa APEXII CCD
Absorption correction	Multi-scan (*SADABS*; Krause *et al.*, 2015 ▸)
*T*_min_, *T*_max_	0.572, 0.653
No. of measured, independent and observed [*I* > 2σ(*I*)] reflections	51429, 8287, 7371
*R*_int_	0.037
(sin θ/λ)_max_ (Å^−1^)	0.707

Refinement	
*R*[*F* ^2^ > 2σ(*F* ^2^)], *wR*(*F* ^2^), *S*	0.033, 0.076, 1.10
No. of reflections	8287
No. of parameters	448
H-atom treatment	H-atom parameters constrained
Δρ_max_, Δρ_min_ (e Å^−3^)	0.81, −0.69

Computer programs: *APEX3* and *SAINT* (Bruker, 2016 ▸), *SHELXS97* (Sheldrick, 2008 ▸), *SHELXL2018/1* (Sheldrick, 2015 ▸), *DIAMOND* (Brandenburg, 2009 ▸) and *publCIF* (Westrip, 2010 ▸).

## supplementary crystallographic information

### Crystal data

**Table:**

[K_4_Ni_2_(C_7_H_6_N_4_O_7_)_2_]·4.8H_2_O	*D*_x_ = 2.053 Mg m^−^^3^
*M**_r_* = 876.66	Mo *K*α radiation, λ = 0.71073 Å
Orthorhombic, *P**b**c**a*	Cell parameters from 18674 reflections
*a* = 15.0694 (3) Å	θ = 1.0–30.0°
*b* = 16.9659 (3) Å	µ = 2.01 mm^−^^1^
*c* = 22.1920 (4) Å	*T* = 100 K
*V* = 5673.74 (18) Å^3^	Block, orange
*Z* = 8	0.31 × 0.26 × 0.23 mm
*F*(000) = 3552	

### Data collection

**Table:**

Bruker Kappa APEXII CCD diffractometer	8287 independent reflections
Radiation source: fine-focus sealed tube	7371 reflections with *I* > 2σ(*I*)
Horizontally mounted graphite crystal monochromator	*R*_int_ = 0.037
Detector resolution: 16 pixels mm^-1^	θ_max_ = 30.2°, θ_min_ = 2.0°
φ scans and ω scans with κ offset	*h* = −20→21
Absorption correction: multi-scan (SADABS; Krause *et al.*, 2015)	*k* = −23→19
*T*_min_ = 0.572, *T*_max_ = 0.653	*l* = −31→31
51429 measured reflections	

### Refinement

**Table:**

Refinement on *F*^2^	0 restraints
Least-squares matrix: full	Hydrogen site location: mixed
*R*[*F*^2^ > 2σ(*F*^2^)] = 0.033	H-atom parameters constrained
*wR*(*F*^2^) = 0.076	*w* = 1/[σ^2^(*F*_o_^2^) + (0.0165*P*)^2^ + 9.9285*P*] where *P* = (*F*_o_^2^ + 2*F*_c_^2^)/3
*S* = 1.10	(Δ/σ)_max_ = 0.002
8287 reflections	Δρ_max_ = 0.81 e Å^−^^3^
448 parameters	Δρ_min_ = −0.69 e Å^−^^3^

### Special details

**Table:**

Geometry. All esds (except the esd in the dihedral angle between two l.s. planes) are estimated using the full covariance matrix. The cell esds are taken into account individually in the estimation of esds in distances, angles and torsion angles; correlations between esds in cell parameters are only used when they are defined by crystal symmetry. An approximate (isotropic) treatment of cell esds is used for estimating esds involving l.s. planes.

### Fractional atomic coordinates and isotropic or equivalent isotropic displacement parameters (Å^2^)

**Table:**

	*x*	*y*	*z*	*U*_iso_*/*U*_eq_	Occ. (<1)
Ni1	0.39976 (2)	0.03560 (2)	0.25909 (2)	0.01144 (6)	
K1	0.42066 (3)	0.10115 (3)	0.50619 (2)	0.01944 (9)	
K2	0.62602 (3)	0.12976 (3)	0.37947 (2)	0.01642 (8)	
K3A	0.6573 (3)	0.0634 (2)	0.0628 (2)	0.0156 (10)	0.46 (3)
K3B	0.6592 (3)	0.0786 (9)	0.0646 (2)	0.0447 (9)	0.54 (3)
K4A	0.81367 (4)	0.18864 (3)	0.48907 (2)	0.02787 (14)	0.9643 (15)
K4B	0.8620 (12)	0.1672 (10)	0.4887 (6)	0.02787 (14)	0.0357 (15)
O1	0.48022 (9)	0.05193 (8)	0.19539 (6)	0.0158 (3)	
O2	0.48968 (8)	0.03838 (8)	0.31915 (6)	0.0147 (3)	
O3	0.50728 (9)	0.01073 (9)	0.41691 (6)	0.0175 (3)	
O4	0.32237 (9)	−0.00551 (9)	0.42549 (6)	0.0187 (3)	
O5	0.23246 (9)	−0.10170 (8)	0.25535 (6)	0.0175 (3)	
O6	0.29549 (9)	0.06429 (10)	0.09851 (6)	0.0209 (3)	
O7	0.48153 (10)	0.07263 (11)	0.09625 (7)	0.0290 (4)	
O1W	0.24931 (12)	0.08231 (11)	0.54478 (7)	0.0294 (4)	
H1W1	0.236269	0.039493	0.568245	0.044*	
H2W1	0.226969	0.122493	0.559745	0.044*	
O2W	0.63486 (14)	0.13337 (10)	0.50382 (8)	0.0361 (4)	
H1W2	0.624292	0.171955	0.530219	0.054*	
H2W2	0.647697	0.086965	0.524420	0.054*	
O3W	0.91669 (13)	0.06558 (12)	0.52097 (8)	0.0383 (4)	
H1W3	0.926510	0.062280	0.482596	0.057*	
H2W3	0.967921	0.084541	0.536516	0.057*	
O4WA	0.60586 (13)	0.23598 (13)	0.07782 (10)	0.0309 (6)	0.805 (4)
H1W4	0.655353	0.218452	0.090399	0.046*	0.805 (4)
H2W4	0.567382	0.239687	0.109145	0.046*	0.805 (4)
O5W	0.5651 (2)	0.16499 (15)	−0.03385 (13)	0.0532 (9)	0.805 (4)
H5WC	0.546573	0.207539	−0.049704	0.080*	0.805 (4)
H5WB	0.588393	0.177329	−0.000284	0.080*	0.805 (4)
O4WB	0.5638 (6)	0.2234 (6)	0.0249 (4)	0.032 (2)	0.195 (4)
H3W4	0.531930	0.234322	0.055384	0.048*	0.195 (4)
H4W4	0.532796	0.240423	−0.006182	0.048*	0.195 (4)
N1	0.32286 (10)	0.01942 (9)	0.32277 (7)	0.0125 (3)	
N2	0.22857 (10)	0.01217 (10)	0.31956 (7)	0.0129 (3)	
N3	0.22075 (10)	0.02637 (10)	0.20888 (7)	0.0137 (3)	
N4	0.31391 (10)	0.03568 (10)	0.19995 (7)	0.0128 (3)	
C1	0.46081 (12)	0.01956 (11)	0.37164 (8)	0.0134 (3)	
C2	0.35945 (12)	0.00948 (11)	0.37637 (8)	0.0131 (3)	
C3	0.19564 (12)	0.05825 (11)	0.26801 (8)	0.0138 (3)	
H3A	0.130103	0.060914	0.270332	0.017*	
H3B	0.218626	0.112739	0.271334	0.017*	
C4	0.20301 (13)	−0.07011 (12)	0.31134 (9)	0.0167 (4)	
H4A	0.228229	−0.101882	0.344615	0.020*	
H4B	0.137568	−0.074362	0.313553	0.020*	
C5	0.19597 (13)	−0.05675 (12)	0.20682 (9)	0.0177 (4)	
H5A	0.130467	−0.061003	0.208006	0.021*	
H5B	0.216417	−0.079534	0.168170	0.021*	
C6	0.34091 (12)	0.05407 (11)	0.14507 (8)	0.0146 (3)	
C7	0.44272 (12)	0.06056 (12)	0.14422 (9)	0.0166 (4)	
Ni1B	0.85260 (2)	0.23735 (2)	0.24199 (2)	0.01304 (6)	
O1B	0.77709 (9)	0.22102 (9)	0.17472 (6)	0.0180 (3)	
O2B	0.75916 (9)	0.23086 (8)	0.29873 (6)	0.0162 (3)	
O3B	0.73206 (9)	0.26008 (9)	0.39495 (7)	0.0212 (3)	
O4B	0.91522 (10)	0.28278 (10)	0.41008 (7)	0.0220 (3)	
O5B	1.01572 (10)	0.37800 (8)	0.24472 (7)	0.0203 (3)	
O6B	0.96972 (11)	0.21209 (10)	0.08487 (7)	0.0253 (3)	
O7B	0.78613 (11)	0.19730 (10)	0.07598 (7)	0.0251 (3)	
N1B	0.92407 (10)	0.25393 (10)	0.30822 (7)	0.0148 (3)	
N2B	1.01790 (10)	0.26347 (10)	0.30814 (7)	0.0151 (3)	
N3B	1.03515 (10)	0.25123 (10)	0.19792 (7)	0.0160 (3)	
N4B	0.94292 (10)	0.23990 (10)	0.18580 (7)	0.0153 (3)	
C1B	0.78284 (12)	0.25187 (11)	0.35171 (9)	0.0152 (3)	
C2B	0.88312 (12)	0.26501 (11)	0.36010 (9)	0.0153 (3)	
C3B	1.05635 (12)	0.21901 (12)	0.25760 (9)	0.0159 (3)	
H3B1	1.121650	0.217835	0.262372	0.019*	
H3B2	1.034773	0.163958	0.259596	0.019*	
C4B	1.04157 (13)	0.34622 (12)	0.30124 (9)	0.0196 (4)	
H4B1	1.012978	0.376994	0.333794	0.024*	
H4B2	1.106608	0.351872	0.305799	0.024*	
C5B	1.05732 (13)	0.33496 (12)	0.19717 (10)	0.0200 (4)	
H5B1	1.122471	0.340939	0.200622	0.024*	
H5B2	1.038927	0.357734	0.158040	0.024*	
C6B	0.92131 (13)	0.22112 (12)	0.13005 (9)	0.0171 (4)	
C7B	0.82017 (13)	0.21177 (12)	0.12558 (9)	0.0178 (4)	

### Atomic displacement parameters (Å^2^)

**Table:**

	*U*^11^	*U*^22^	*U*^33^	*U*^12^	*U*^13^	*U*^23^
Ni1	0.00784 (10)	0.01467 (12)	0.01179 (11)	−0.00082 (8)	−0.00019 (7)	0.00124 (8)
K1	0.0243 (2)	0.0196 (2)	0.01449 (18)	0.00216 (16)	−0.00152 (15)	−0.00092 (15)
K2	0.01387 (17)	0.0187 (2)	0.01671 (18)	−0.00075 (14)	0.00052 (14)	0.00027 (14)
K3A	0.0119 (9)	0.018 (2)	0.0172 (9)	0.0053 (6)	−0.0008 (6)	−0.0050 (9)
K3B	0.0199 (9)	0.083 (3)	0.0313 (13)	−0.0053 (16)	−0.0054 (8)	0.0187 (14)
K4A	0.0356 (3)	0.0307 (3)	0.0173 (2)	0.0017 (2)	−0.00088 (19)	−0.00179 (19)
K4B	0.0356 (3)	0.0307 (3)	0.0173 (2)	0.0017 (2)	−0.00088 (19)	−0.00179 (19)
O1	0.0110 (6)	0.0210 (7)	0.0154 (6)	−0.0016 (5)	0.0011 (5)	0.0024 (5)
O2	0.0110 (6)	0.0193 (7)	0.0138 (6)	−0.0006 (5)	−0.0022 (5)	0.0009 (5)
O3	0.0155 (6)	0.0211 (7)	0.0160 (6)	0.0007 (5)	−0.0042 (5)	0.0005 (5)
O4	0.0162 (6)	0.0268 (8)	0.0132 (6)	0.0031 (6)	0.0017 (5)	0.0019 (5)
O5	0.0177 (7)	0.0146 (7)	0.0203 (7)	−0.0005 (5)	−0.0007 (5)	0.0000 (5)
O6	0.0153 (6)	0.0338 (9)	0.0136 (6)	−0.0009 (6)	−0.0015 (5)	0.0034 (6)
O7	0.0168 (7)	0.0527 (11)	0.0176 (7)	−0.0055 (7)	0.0049 (6)	0.0025 (7)
O1W	0.0343 (9)	0.0321 (9)	0.0219 (7)	−0.0018 (7)	0.0047 (7)	−0.0029 (7)
O2W	0.0642 (13)	0.0242 (9)	0.0199 (8)	0.0043 (8)	−0.0021 (8)	0.0021 (6)
O3W	0.0388 (10)	0.0525 (12)	0.0236 (8)	0.0080 (9)	0.0036 (7)	0.0022 (8)
O4WA	0.0185 (9)	0.0401 (13)	0.0341 (12)	0.0043 (8)	0.0101 (8)	0.0022 (9)
O5W	0.073 (2)	0.0320 (14)	0.0542 (17)	−0.0034 (13)	−0.0292 (15)	0.0080 (12)
O4WB	0.022 (4)	0.039 (5)	0.035 (5)	0.010 (4)	0.003 (3)	0.012 (4)
N1	0.0084 (6)	0.0152 (8)	0.0139 (7)	0.0007 (5)	0.0009 (5)	0.0004 (6)
N2	0.0081 (6)	0.0157 (8)	0.0150 (7)	−0.0004 (5)	0.0000 (5)	0.0016 (6)
N3	0.0072 (6)	0.0181 (8)	0.0158 (7)	−0.0021 (5)	0.0002 (5)	0.0014 (6)
N4	0.0083 (6)	0.0167 (8)	0.0133 (7)	−0.0014 (5)	0.0004 (5)	0.0021 (6)
C1	0.0137 (8)	0.0108 (8)	0.0159 (8)	0.0015 (6)	−0.0011 (6)	−0.0014 (6)
C2	0.0123 (8)	0.0126 (8)	0.0143 (8)	0.0026 (6)	0.0006 (6)	0.0001 (6)
C3	0.0103 (7)	0.0153 (9)	0.0159 (8)	0.0008 (6)	0.0004 (6)	0.0022 (6)
C4	0.0146 (8)	0.0171 (9)	0.0184 (9)	−0.0031 (7)	0.0008 (7)	0.0036 (7)
C5	0.0157 (8)	0.0192 (10)	0.0183 (9)	−0.0039 (7)	−0.0008 (7)	−0.0002 (7)
C6	0.0120 (8)	0.0169 (9)	0.0151 (8)	−0.0006 (6)	−0.0002 (6)	0.0008 (7)
C7	0.0139 (8)	0.0190 (10)	0.0169 (8)	−0.0011 (7)	0.0014 (7)	0.0004 (7)
Ni1B	0.00973 (10)	0.01497 (12)	0.01442 (11)	−0.00085 (8)	−0.00058 (8)	−0.00124 (8)
O1B	0.0152 (6)	0.0210 (7)	0.0179 (6)	−0.0014 (5)	−0.0030 (5)	−0.0007 (5)
O2B	0.0114 (6)	0.0196 (7)	0.0175 (6)	−0.0014 (5)	−0.0002 (5)	−0.0011 (5)
O3B	0.0159 (6)	0.0268 (8)	0.0210 (7)	−0.0019 (6)	0.0040 (5)	−0.0030 (6)
O4B	0.0181 (7)	0.0302 (8)	0.0177 (7)	−0.0016 (6)	−0.0019 (5)	−0.0070 (6)
O5B	0.0196 (7)	0.0133 (7)	0.0280 (8)	−0.0002 (5)	0.0034 (6)	−0.0014 (6)
O6B	0.0247 (8)	0.0331 (9)	0.0180 (7)	−0.0001 (6)	0.0047 (6)	−0.0061 (6)
O7B	0.0276 (8)	0.0291 (9)	0.0186 (7)	−0.0036 (6)	−0.0072 (6)	0.0003 (6)
N1B	0.0099 (7)	0.0166 (8)	0.0178 (7)	−0.0002 (6)	−0.0007 (5)	−0.0028 (6)
N2B	0.0091 (6)	0.0160 (8)	0.0202 (8)	−0.0001 (6)	−0.0006 (6)	−0.0044 (6)
N3B	0.0118 (7)	0.0170 (8)	0.0191 (8)	−0.0014 (6)	0.0017 (6)	−0.0022 (6)
N4B	0.0122 (7)	0.0154 (8)	0.0183 (7)	−0.0016 (6)	0.0004 (6)	−0.0021 (6)
C1B	0.0144 (8)	0.0139 (9)	0.0172 (8)	−0.0006 (7)	0.0011 (6)	−0.0004 (7)
C2B	0.0136 (8)	0.0146 (9)	0.0178 (8)	−0.0006 (6)	0.0001 (6)	−0.0016 (7)
C3B	0.0109 (8)	0.0144 (9)	0.0224 (9)	0.0018 (6)	0.0005 (6)	−0.0024 (7)
C4B	0.0153 (8)	0.0188 (10)	0.0248 (10)	−0.0027 (7)	0.0010 (7)	−0.0064 (8)
C5B	0.0158 (9)	0.0186 (10)	0.0255 (10)	−0.0024 (7)	0.0047 (7)	−0.0002 (8)
C6B	0.0187 (9)	0.0143 (9)	0.0183 (9)	−0.0002 (7)	0.0008 (7)	−0.0009 (7)
C7B	0.0210 (9)	0.0131 (9)	0.0193 (9)	−0.0012 (7)	−0.0034 (7)	−0.0003 (7)

### Geometric parameters (Å, º)

**Table:**

Ni1—N4	1.8429 (15)	K4B—O3B	3.263 (17)
Ni1—N1	1.8479 (15)	K4B—C2B	3.316 (14)
Ni1—O1	1.8830 (13)	K4B—O6B^vi^	3.374 (15)
Ni1—O2	1.9012 (13)	O1—C7	1.277 (2)
K1—O4B^i^	2.7086 (15)	O2—C1	1.284 (2)
K1—O1W	2.7392 (18)	O3—C1	1.234 (2)
K1—O3^ii^	2.7739 (14)	O4—C2	1.251 (2)
K1—O3	2.8254 (15)	O5—C4	1.424 (2)
K1—O6B^iii^	2.8588 (16)	O5—C5	1.430 (2)
K1—O4	2.9456 (15)	O6—C6	1.252 (2)
K1—O7B^iii^	3.1774 (17)	O7—C7	1.232 (2)
K1—O2W	3.274 (2)	O1W—H1W1	0.9152
K1—C1	3.3464 (19)	O1W—H2W1	0.8297
K1—C2	3.4015 (19)	O2W—H1W2	0.8929
K1—K4A^i^	3.9153 (7)	O2W—H2W2	0.9306
K1—K4B^i^	4.030 (16)	O3W—H1W3	0.8662
K2—O3B	2.7495 (16)	O3W—H2W3	0.9048
K2—O2W	2.7634 (17)	O4WA—H1W4	0.8501
K2—O3	2.8232 (15)	O4WA—H2W4	0.9075
K2—O6^iv^	2.8273 (15)	O5W—H5WC	0.8499
K2—O6B^iii^	2.8505 (17)	O5W—H5WB	0.8499
K2—O2	2.9013 (14)	O4WB—H3W4	0.8501
K2—N3^iv^	2.9932 (16)	O4WB—H4W4	0.8827
K2—N3B^iii^	3.0121 (17)	N1—C2	1.322 (2)
K2—C1	3.1184 (19)	N1—N2	1.428 (2)
K2—O2B	3.1903 (14)	N2—C4	1.460 (2)
K2—C1B	3.2025 (19)	N2—C3	1.472 (2)
K2—C6B^iii^	3.459 (2)	N3—N4	1.427 (2)
K3A—O3W^v^	2.625 (4)	N3—C5	1.460 (3)
K3A—O7	2.755 (4)	N3—C3	1.469 (2)
K3A—O4^iv^	2.761 (4)	N4—C6	1.321 (2)
K3A—O1W^iv^	2.779 (5)	C1—C2	1.541 (2)
K3A—N2^iv^	2.954 (5)	C3—H3A	0.9900
K3A—O7B	3.003 (5)	C3—H3B	0.9900
K3A—O4WA	3.047 (4)	C4—H4A	0.9900
K3A—O5W	3.082 (5)	C4—H4B	0.9900
K3A—O4WB	3.171 (9)	C5—H5A	0.9900
K3A—C2^iv^	3.456 (5)	C5—H5B	0.9900
K3A—K4B^v^	4.254 (16)	C6—C7	1.538 (3)
K3B—O7	2.769 (5)	Ni1B—N1B	1.8436 (16)
K3B—O1W^iv^	2.783 (5)	Ni1B—N4B	1.8463 (16)
K3B—O7B	2.789 (13)	Ni1B—O2B	1.8922 (14)
K3B—O4WA	2.804 (15)	Ni1B—O1B	1.8975 (14)
K3B—O4^iv^	2.851 (8)	O1B—C7B	1.279 (2)
K3B—O3W^v^	2.868 (15)	O2B—C1B	1.279 (2)
K3B—O4WB	2.979 (15)	O3B—C1B	1.235 (2)
K3B—O5W	2.990 (7)	O4B—C2B	1.247 (2)
K3B—N2^iv^	2.995 (6)	O5B—C4B	1.420 (3)
K3B—C2^iv^	3.492 (6)	O5B—C5B	1.428 (2)
K3B—K4B^v^	4.51 (2)	O6B—C6B	1.249 (2)
K4A—K4B	0.815 (18)	O7B—C7B	1.239 (2)
K4A—O3W	2.696 (2)	N1B—C2B	1.320 (2)
K4A—O3B	2.7100 (16)	N1B—N2B	1.423 (2)
K4A—O7B^vi^	2.7633 (17)	N2B—C4B	1.457 (3)
K4A—O4B	2.8223 (17)	N2B—C3B	1.471 (2)
K4A—O2W	2.872 (2)	N3B—N4B	1.429 (2)
K4A—O6^iv^	2.8815 (16)	N3B—C5B	1.459 (3)
K4A—C1B	3.265 (2)	N3B—C3B	1.468 (3)
K4A—C2B	3.311 (2)	N4B—C6B	1.318 (2)
K4A—C7B^vi^	3.470 (2)	C1B—C2B	1.539 (3)
K4B—O3W	2.040 (17)	C3B—H3B1	0.9900
K4B—O4B	2.744 (15)	C3B—H3B2	0.9900
K4B—O6^iv^	2.792 (14)	C4B—H4B1	0.9900
K4B—O7^iv^	3.061 (17)	C4B—H4B2	0.9900
K4B—O4WB^iv^	3.201 (19)	C5B—H5B1	0.9900
K4B—O7B^vi^	3.216 (16)	C5B—H5B2	0.9900
K4B—O5W^iv^	3.220 (18)	C6B—C7B	1.536 (3)
			
N4—Ni1—N1	96.01 (7)	O3W—K4A—C7B^vi^	97.56 (5)
N4—Ni1—O1	85.25 (6)	O3B—K4A—C7B^vi^	117.90 (5)
N1—Ni1—O1	178.74 (6)	O7B^vi^—K4A—C7B^vi^	18.91 (4)
N4—Ni1—O2	178.28 (7)	O4B—K4A—C7B^vi^	104.56 (5)
N1—Ni1—O2	85.10 (6)	O2W—K4A—C7B^vi^	94.93 (5)
O1—Ni1—O2	93.65 (6)	O6^iv^—K4A—C7B^vi^	161.44 (5)
O4B^i^—K1—O1W	80.90 (5)	C1B—K4A—C7B^vi^	131.24 (5)
O4B^i^—K1—O3^ii^	95.01 (5)	C2B—K4A—C7B^vi^	123.72 (5)
O1W—K1—O3^ii^	95.56 (5)	O3W—K4A—K2	112.75 (5)
O4B^i^—K1—O3	152.85 (5)	O3B—K4A—K2	45.40 (3)
O1W—K1—O3	126.25 (5)	O7B^vi^—K4A—K2	120.72 (4)
O3^ii^—K1—O3	83.05 (4)	O4B—K4A—K2	98.76 (3)
O4B^i^—K1—O6B^iii^	90.81 (5)	O2W—K4A—K2	45.59 (3)
O1W—K1—O6B^iii^	122.79 (5)	O6^iv^—K4A—K2	46.85 (3)
O3^ii^—K1—O6B^iii^	141.65 (5)	C1B—K4A—K2	52.60 (3)
O3—K1—O6B^iii^	75.07 (5)	C2B—K4A—K2	77.78 (3)
O4B^i^—K1—O4	148.05 (5)	C7B^vi^—K4A—K2	134.15 (4)
O1W—K1—O4	69.17 (4)	O3W—K4A—K1^vii^	117.46 (5)
O3^ii^—K1—O4	98.66 (4)	O3B—K4A—K1^vii^	78.47 (4)
O3—K1—O4	58.15 (4)	O7B^vi^—K4A—K1^vii^	53.51 (4)
O6B^iii^—K1—O4	96.01 (4)	O4B—K4A—K1^vii^	43.77 (3)
O4B^i^—K1—O7B^iii^	90.06 (5)	O2W—K4A—K1^vii^	132.91 (4)
O1W—K1—O7B^iii^	68.77 (5)	O6^iv^—K4A—K1^vii^	136.40 (3)
O3^ii^—K1—O7B^iii^	162.58 (4)	C1B—K4A—K1^vii^	77.55 (4)
O3—K1—O7B^iii^	99.85 (4)	C2B—K4A—K1^vii^	62.40 (3)
O6B^iii^—K1—O7B^iii^	54.64 (4)	C7B^vi^—K4A—K1^vii^	61.41 (4)
O4—K1—O7B^iii^	69.24 (4)	K2—K4A—K1^vii^	123.652 (17)
O4B^i^—K1—O2W	85.38 (5)	O3W—K4B—O4B	135.2 (8)
O1W—K1—O2W	162.52 (5)	O3W—K4B—O6^iv^	82.0 (5)
O3^ii^—K1—O2W	74.82 (4)	O4B—K4B—O6^iv^	96.4 (4)
O3—K1—O2W	67.91 (4)	O3W—K4B—O7^iv^	62.4 (4)
O6B^iii^—K1—O2W	67.90 (4)	O4B—K4B—O7^iv^	79.1 (4)
O4—K1—O2W	126.05 (4)	O6^iv^—K4B—O7^iv^	57.1 (3)
O7B^iii^—K1—O2W	122.28 (4)	O3W—K4B—O4WB^iv^	84.3 (6)
O4B^i^—K1—C1	156.68 (5)	O4B—K4B—O4WB^iv^	56.6 (4)
O1W—K1—C1	113.65 (5)	O6^iv^—K4B—O4WB^iv^	117.5 (5)
O3^ii^—K1—C1	101.25 (5)	O7^iv^—K4B—O4WB^iv^	62.6 (4)
O3—K1—C1	20.96 (4)	O3W—K4B—O7B^vi^	122.4 (5)
O6B^iii^—K1—C1	66.10 (5)	O4B—K4B—O7B^vi^	88.6 (4)
O4—K1—C1	45.11 (4)	O6^iv^—K4B—O7B^vi^	137.4 (7)
O7B^iii^—K1—C1	79.38 (4)	O7^iv^—K4B—O7B^vi^	162.7 (6)
O2W—K1—C1	82.91 (4)	O3W—K4B—O5W^iv^	59.8 (4)
O4B^i^—K1—C2	154.85 (5)	O4B—K4B—O5W^iv^	85.9 (4)
O1W—K1—C2	87.46 (5)	O6^iv^—K4B—O5W^iv^	123.3 (6)
O3^ii^—K1—C2	108.32 (4)	O7^iv^—K4B—O5W^iv^	68.1 (4)
O3—K1—C2	44.20 (4)	O7B^vi^—K4B—O5W^iv^	99.1 (4)
O6B^iii^—K1—C2	76.85 (5)	O3W—K4B—O3B	150.9 (7)
O4—K1—C2	21.21 (4)	O4B—K4B—O3B	54.9 (3)
O7B^iii^—K1—C2	64.89 (4)	O6^iv^—K4B—O3B	69.2 (3)
O2W—K1—C2	109.27 (4)	O7^iv^—K4B—O3B	102.3 (4)
C1—K1—C2	26.38 (4)	O4WB^iv^—K4B—O3B	111.5 (5)
O4B^i^—K1—K4A^i^	46.13 (3)	O7B^vi^—K4B—O3B	79.9 (4)
O1W—K1—K4A^i^	73.12 (4)	O5W^iv^—K4B—O3B	140.8 (5)
O3^ii^—K1—K4A^i^	140.17 (3)	O3W—K4B—C2B	133.3 (7)
O3—K1—K4A^i^	134.72 (3)	O4B—K4B—C2B	21.17 (12)
O6B^iii^—K1—K4A^i^	61.69 (4)	O6^iv^—K4B—C2B	75.6 (3)
O4—K1—K4A^i^	111.64 (3)	O7^iv^—K4B—C2B	71.1 (3)
O7B^iii^—K1—K4A^i^	44.36 (3)	O4WB^iv^—K4B—C2B	71.3 (4)
O2W—K1—K4A^i^	104.74 (3)	O7B^vi^—K4B—C2B	101.2 (5)
C1—K1—K4A^i^	118.37 (3)	O5W^iv^—K4B—C2B	100.5 (4)
C2—K1—K4A^i^	109.12 (3)	O3B—K4B—C2B	42.9 (2)
O4B^i^—K1—K4B^i^	42.7 (2)	O3W—K4B—O6B^vi^	95.5 (5)
O1W—K1—K4B^i^	84.2 (3)	O4B—K4B—O6B^vi^	80.1 (4)
O3^ii^—K1—K4B^i^	137.4 (2)	O6^iv^—K4B—O6B^vi^	172.1 (7)
O3—K1—K4B^i^	130.6 (2)	O7^iv^—K4B—O6B^vi^	115.1 (5)
O6B^iii^—K1—K4B^i^	55.6 (2)	O7B^vi^—K4B—O6B^vi^	49.9 (2)
O4—K1—K4B^i^	120.4 (2)	O5W^iv^—K4B—O6B^vi^	49.7 (2)
O7B^iii^—K1—K4B^i^	51.4 (2)	O3B—K4B—O6B^vi^	113.5 (5)
O2W—K1—K4B^i^	93.1 (3)	C2B—K4B—O6B^vi^	101.2 (4)
C1—K1—K4B^i^	117.9 (2)	O3W—K4B—K1^vii^	136.5 (6)
C2—K1—K4B^i^	114.2 (2)	O4B—K4B—K1^vii^	42.0 (2)
K4A^i^—K1—K4B^i^	11.7 (3)	O6^iv^—K4B—K1^vii^	135.1 (5)
O3B—K2—O2W	80.18 (5)	O7^iv^—K4B—K1^vii^	113.5 (4)
O3B—K2—O3	155.07 (4)	O7B^vi^—K4B—K1^vii^	50.5 (2)
O2W—K2—O3	75.67 (5)	O5W^iv^—K4B—K1^vii^	78.1 (3)
O3B—K2—O6^iv^	76.67 (5)	O3B—K4B—K1^vii^	71.2 (3)
O2W—K2—O6^iv^	78.03 (5)	C2B—K4B—K1^vii^	61.0 (3)
O3—K2—O6^iv^	103.92 (4)	O6B^vi^—K4B—K1^vii^	44.33 (19)
O3B—K2—O6B^iii^	92.95 (5)	C7—O1—Ni1	113.55 (12)
O2W—K2—O6B^iii^	75.63 (5)	C1—O2—Ni1	112.85 (11)
O3—K2—O6B^iii^	75.23 (5)	C1—O2—K2	87.38 (10)
O6^iv^—K2—O6B^iii^	152.98 (4)	Ni1—O2—K2	147.29 (7)
O3B—K2—O2	154.02 (4)	C1—O3—K1^ii^	143.74 (13)
O2W—K2—O2	120.44 (5)	C1—O3—K2	91.90 (11)
O3—K2—O2	45.94 (4)	K1^ii^—O3—K2	114.99 (5)
O6^iv^—K2—O2	120.63 (4)	C1—O3—K1	104.05 (12)
O6B^iii^—K2—O2	78.74 (4)	K1^ii^—O3—K1	96.95 (4)
O3B—K2—N3^iv^	106.02 (4)	K2—O3—K1	96.36 (5)
O2W—K2—N3^iv^	130.10 (5)	C2—O4—K3A^iii^	113.48 (14)
O3—K2—N3^iv^	94.35 (4)	C2—O4—K3B^iii^	110.5 (2)
O6^iv^—K2—N3^iv^	56.79 (4)	C2—O4—K1	100.39 (12)
O6B^iii^—K2—N3^iv^	149.67 (5)	K3A^iii^—O4—K1	97.80 (11)
O2—K2—N3^iv^	73.88 (4)	K3B^iii^—O4—K1	94.6 (2)
O3B—K2—N3B^iii^	77.60 (5)	C4—O5—C5	109.67 (15)
O2W—K2—N3B^iii^	125.18 (5)	C6—O6—K4B^iii^	117.5 (4)
O3—K2—N3B^iii^	111.66 (4)	C6—O6—K2^iii^	113.93 (12)
O6^iv^—K2—N3B^iii^	141.08 (5)	K4B^iii^—O6—K2^iii^	101.5 (4)
O6B^iii^—K2—N3B^iii^	56.45 (4)	C6—O6—K4A^iii^	127.32 (13)
O2—K2—N3B^iii^	77.33 (4)	K2^iii^—O6—K4A^iii^	85.11 (4)
N3^iv^—K2—N3B^iii^	104.14 (5)	C7—O7—K3A	132.83 (17)
O3B—K2—C1	162.38 (5)	C7—O7—K3B	133.15 (17)
O2W—K2—C1	96.17 (6)	C7—O7—K4B^iii^	109.9 (3)
O3—K2—C1	23.29 (4)	K3A—O7—K4B^iii^	115.4 (3)
O6^iv^—K2—C1	119.69 (5)	K3B—O7—K4B^iii^	113.2 (3)
O6B^iii^—K2—C1	69.48 (5)	K1—O1W—K3A^iii^	102.44 (11)
O2—K2—C1	24.28 (4)	K1—O1W—K3B^iii^	100.96 (14)
N3^iv^—K2—C1	89.59 (5)	K1—O1W—H1W1	118.2
N3B^iii^—K2—C1	90.89 (5)	K3A^iii^—O1W—H1W1	106.9
O3B—K2—O2B	43.31 (4)	K3B^iii^—O1W—H1W1	111.9
O2W—K2—O2B	121.24 (5)	K1—O1W—H2W1	114.3
O3—K2—O2B	161.52 (4)	K3A^iii^—O1W—H2W1	103.7
O6^iv^—K2—O2B	74.95 (4)	K3B^iii^—O1W—H2W1	99.8
O6B^iii^—K2—O2B	114.34 (4)	H1W1—O1W—H2W1	109.7
O2—K2—O2B	118.26 (4)	K2—O2W—K4A	86.48 (5)
N3^iv^—K2—O2B	69.34 (4)	K2—O2W—K1	87.99 (5)
N3B^iii^—K2—O2B	66.28 (4)	K4A—O2W—K1	168.91 (7)
C1—K2—O2B	142.47 (4)	K2—O2W—H1W2	131.5
O3B—K2—C1B	22.33 (4)	K4A—O2W—H1W2	90.2
O2W—K2—C1B	98.18 (6)	K1—O2W—H1W2	86.3
O3—K2—C1B	170.77 (5)	K2—O2W—H2W2	118.8
O6^iv^—K2—C1B	67.72 (5)	K4A—O2W—H2W2	97.9
O6B^iii^—K2—C1B	110.25 (5)	K1—O2W—H2W2	93.2
O2—K2—C1B	141.22 (5)	H1W2—O2W—H2W2	109.6
N3^iv^—K2—C1B	84.33 (5)	K4B—O3W—K3A^viii^	131.1 (5)
N3B^iii^—K2—C1B	77.48 (5)	K3A^viii^—O3W—K4A	119.59 (11)
C1—K2—C1B	165.07 (5)	K4B—O3W—K3B^viii^	132.7 (5)
O2B—K2—C1B	23.09 (4)	K4A—O3W—K3B^viii^	121.30 (15)
O3B—K2—C6B^iii^	99.54 (5)	K4B—O3W—H1W3	77.2
O2W—K2—C6B^iii^	95.40 (5)	K3A^viii^—O3W—H1W3	111.5
O3—K2—C6B^iii^	76.89 (4)	K4A—O3W—H1W3	83.7
O6^iv^—K2—C6B^iii^	172.82 (5)	K3B^viii^—O3W—H1W3	110.2
O6B^iii^—K2—C6B^iii^	20.01 (4)	K4B—O3W—H2W3	100.3
O2—K2—C6B^iii^	65.14 (4)	K3A^viii^—O3W—H2W3	121.6
N3^iv^—K2—C6B^iii^	130.38 (5)	K4A—O3W—H2W3	108.4
N3B^iii^—K2—C6B^iii^	41.68 (4)	K3B^viii^—O3W—H2W3	121.0
C1—K2—C6B^iii^	63.46 (5)	H1W3—O3W—H2W3	104.6
O2B—K2—C6B^iii^	106.60 (4)	K3B—O4WA—H1W4	56.6
C1B—K2—C6B^iii^	110.89 (5)	K3A—O4WA—H1W4	58.4
O3W^v^—K3A—O7	74.60 (13)	K3B—O4WA—H2W4	109.2
O3W^v^—K3A—O4^iv^	93.60 (12)	K3A—O4WA—H2W4	108.2
O7—K3A—O4^iv^	149.9 (2)	H1W4—O4WA—H2W4	109.5
O3W^v^—K3A—O1W^iv^	90.25 (12)	K3B—O5W—K4B^iii^	103.2 (3)
O7—K3A—O1W^iv^	134.80 (18)	K3B—O5W—H5WC	151.0
O4^iv^—K3A—O1W^iv^	71.35 (10)	K3A—O5W—H5WC	155.6
O3W^v^—K3A—N2^iv^	102.80 (15)	K4B^iii^—O5W—H5WC	78.9
O7—K3A—N2^iv^	97.36 (14)	K3A—O5W—H5WB	48.8
O4^iv^—K3A—N2^iv^	57.62 (10)	K4B^iii^—O5W—H5WB	96.7
O1W^iv^—K3A—N2^iv^	127.71 (15)	H5WC—O5W—H5WB	106.8
O3W^v^—K3A—O7B	159.98 (16)	K3B—O4WB—K4B^iii^	103.9 (5)
O7—K3A—O7B	123.54 (12)	K3A—O4WB—K4B^iii^	101.1 (4)
O4^iv^—K3A—O7B	74.25 (12)	K3B—O4WB—H3W4	102.6
O1W^iv^—K3A—O7B	70.95 (12)	K3A—O4WB—H3W4	103.2
N2^iv^—K3A—O7B	84.34 (12)	K4B^iii^—O4WB—H3W4	66.6
O3W^v^—K3A—O4WA	137.03 (17)	K3B—O4WB—H4W4	139.2
O7—K3A—O4WA	70.82 (9)	K3A—O4WB—H4W4	136.3
O4^iv^—K3A—O4WA	128.73 (16)	K4B^iii^—O4WB—H4W4	61.3
O1W^iv^—K3A—O4WA	96.31 (15)	H3W4—O4WB—H4W4	104.5
N2^iv^—K3A—O4WA	106.17 (12)	C2—N1—N2	116.68 (15)
O7B—K3A—O4WA	55.03 (8)	C2—N1—Ni1	116.46 (12)
O3W^v^—K3A—O5W	91.65 (14)	N2—N1—Ni1	126.77 (12)
O7—K3A—O5W	73.88 (12)	N1—N2—C4	110.56 (14)
O4^iv^—K3A—O5W	135.11 (18)	N1—N2—C3	109.18 (14)
O1W^iv^—K3A—O5W	64.08 (12)	C4—N2—C3	108.78 (15)
N2^iv^—K3A—O5W	160.64 (13)	N1—N2—K3A^iii^	107.01 (13)
O7B—K3A—O5W	86.35 (13)	C4—N2—K3A^iii^	107.21 (13)
O4WA—K3A—O5W	54.85 (10)	C3—N2—K3A^iii^	114.08 (12)
O3W^v^—K3A—O4WB	115.5 (2)	N1—N2—K3B^iii^	105.80 (15)
O7—K3A—O4WB	66.17 (18)	C4—N2—K3B^iii^	112.0 (3)
O4^iv^—K3A—O4WB	142.0 (2)	C3—N2—K3B^iii^	110.5 (3)
O1W^iv^—K3A—O4WB	84.0 (2)	N4—N3—C5	110.76 (15)
N2^iv^—K3A—O4WB	130.4 (2)	N4—N3—C3	109.68 (14)
O7B—K3A—O4WB	70.46 (19)	C5—N3—C3	108.53 (15)
O3W^v^—K3A—C2^iv^	106.96 (13)	N4—N3—K2^iii^	108.26 (10)
O7—K3A—C2^iv^	139.22 (17)	C5—N3—K2^iii^	115.07 (11)
O4^iv^—K3A—C2^iv^	19.39 (5)	C3—N3—K2^iii^	104.28 (10)
O1W^iv^—K3A—C2^iv^	85.77 (11)	C6—N4—N3	117.21 (15)
N2^iv^—K3A—C2^iv^	41.94 (7)	C6—N4—Ni1	116.14 (12)
O7B—K3A—C2^iv^	65.93 (10)	N3—N4—Ni1	126.28 (12)
O4WA—K3A—C2^iv^	115.84 (13)	O3—C1—O2	125.22 (17)
O5W—K3A—C2^iv^	144.82 (17)	O3—C1—C2	119.59 (17)
O3W^v^—K3A—K4B^v^	21.2 (3)	O2—C1—C2	115.18 (15)
O7—K3A—K4B^v^	95.2 (3)	O3—C1—K2	64.80 (10)
O4^iv^—K3A—K4B^v^	73.1 (3)	O2—C1—K2	68.34 (10)
O1W^iv^—K3A—K4B^v^	78.9 (2)	C2—C1—K2	148.69 (12)
N2^iv^—K3A—K4B^v^	95.5 (2)	O3—C1—K1	54.99 (10)
O7B—K3A—K4B^v^	141.0 (3)	O2—C1—K1	140.18 (13)
O4WA—K3A—K4B^v^	155.3 (3)	C2—C1—K1	78.81 (10)
O5W—K3A—K4B^v^	102.4 (2)	K2—C1—K1	81.17 (4)
C2^iv^—K3A—K4B^v^	88.2 (3)	O4—C2—N1	128.58 (17)
O3W^v^—K3A—K1^iv^	112.95 (11)	O4—C2—C1	121.66 (16)
O7—K3A—K1^iv^	167.00 (15)	N1—C2—C1	109.76 (15)
O4^iv^—K3A—K1^iv^	42.72 (7)	O4—C2—K1	58.41 (10)
O1W^iv^—K3A—K1^iv^	38.45 (7)	N1—C2—K1	144.78 (13)
N2^iv^—K3A—K1^iv^	91.31 (11)	C1—C2—K1	74.82 (10)
O7B—K3A—K1^iv^	47.58 (7)	O4—C2—K3A^iii^	47.13 (11)
O4WA—K3A—K1^iv^	97.50 (12)	N1—C2—K3A^iii^	87.14 (12)
O5W—K3A—K1^iv^	94.88 (13)	C1—C2—K3A^iii^	150.62 (14)
C2^iv^—K3A—K1^iv^	50.58 (6)	K1—C2—K3A^iii^	77.71 (9)
K4B^v^—K3A—K1^iv^	93.6 (2)	O4—C2—K3B^iii^	49.9 (2)
O3W^v^—K3A—H1W4	152.5	N1—C2—K3B^iii^	86.22 (14)
O7—K3A—H1W4	82.7	C1—C2—K3B^iii^	147.7 (2)
O4^iv^—K3A—H1W4	113.6	K1—C2—K3B^iii^	76.31 (12)
O1W^iv^—K3A—H1W4	95.0	N3—C3—N2	114.33 (15)
N2^iv^—K3A—H1W4	95.2	N3—C3—H3A	108.7
O7B—K3A—H1W4	41.2	N2—C3—H3A	108.7
O4WA—K3A—H1W4	15.6	N3—C3—H3B	108.7
O5W—K3A—H1W4	66.9	N2—C3—H3B	108.7
C2^iv^—K3A—H1W4	100.3	H3A—C3—H3B	107.6
K4B^v^—K3A—H1W4	169.3	O5—C4—N2	112.75 (15)
K1^iv^—K3A—H1W4	86.9	O5—C4—H4A	109.0
O3W^v^—K3A—H5WB	105.0	N2—C4—H4A	109.0
O7—K3A—H5WB	73.7	O5—C4—H4B	109.0
O4^iv^—K3A—H5WB	136.4	N2—C4—H4B	109.0
O1W^iv^—K3A—H5WB	69.6	H4A—C4—H4B	107.8
N2^iv^—K3A—H5WB	147.0	O5—C5—N3	113.17 (15)
O7B—K3A—H5WB	75.4	O5—C5—H5A	108.9
O4WA—K3A—H5WB	40.9	N3—C5—H5A	108.9
O5W—K3A—H5WB	14.2	O5—C5—H5B	108.9
C2^iv^—K3A—H5WB	139.3	N3—C5—H5B	108.9
K4B^v^—K3A—H5WB	116.6	H5A—C5—H5B	107.8
K1^iv^—K3A—H5WB	93.8	O6—C6—N4	128.70 (17)
H1W4—K3A—H5WB	52.7	O6—C6—C7	121.70 (17)
O7—K3B—O1W^iv^	133.9 (2)	N4—C6—C7	109.59 (16)
O7—K3B—O7B	131.8 (5)	O6—C6—K2^iii^	47.14 (10)
O1W^iv^—K3B—O7B	74.2 (2)	N4—C6—K2^iii^	86.88 (11)
O7—K3B—O4WA	74.4 (2)	C7—C6—K2^iii^	152.63 (13)
O1W^iv^—K3B—O4WA	102.1 (4)	O7—C7—O1	125.28 (18)
O7B—K3B—O4WA	60.0 (3)	O7—C7—C6	119.73 (17)
O7—K3B—O4^iv^	142.7 (5)	O1—C7—C6	114.98 (16)
O1W^iv^—K3B—O4^iv^	69.98 (15)	N1B—Ni1B—N4B	95.98 (7)
O7B—K3B—O4^iv^	76.28 (15)	N1B—Ni1B—O2B	85.01 (6)
O4WA—K3B—O4^iv^	135.7 (5)	N4B—Ni1B—O2B	177.89 (7)
O7—K3B—O3W^v^	70.7 (3)	N1B—Ni1B—O1B	178.86 (7)
O1W^iv^—K3B—O3W^v^	85.4 (3)	N4B—Ni1B—O1B	85.08 (7)
O7B—K3B—O3W^v^	156.9 (3)	O2B—Ni1B—O1B	93.94 (6)
O4WA—K3B—O3W^v^	137.15 (18)	C7B—O1B—Ni1B	112.61 (12)
O4^iv^—K3B—O3W^v^	86.7 (4)	C1B—O2B—Ni1B	112.81 (12)
O7—K3B—O4WB	68.8 (2)	C1B—O2B—K2	78.99 (10)
O1W^iv^—K3B—O4WB	87.6 (3)	Ni1B—O2B—K2	150.81 (7)
O7B—K3B—O4WB	76.2 (4)	C1B—O3B—K4A	105.49 (12)
O4^iv^—K3B—O4WB	148.3 (4)	C1B—O3B—K2	99.92 (12)
O3W^v^—K3B—O4WB	114.3 (3)	K4A—O3B—K2	90.02 (5)
O7—K3B—O5W	75.20 (14)	C1B—O3B—K4B	93.9 (3)
O1W^iv^—K3B—O5W	65.33 (14)	K2—O3B—K4B	92.3 (3)
O7B—K3B—O5W	92.1 (4)	C2B—O4B—K1^vii^	142.90 (14)
O4WA—K3B—O5W	58.2 (2)	C2B—O4B—K4B	106.2 (3)
O4^iv^—K3B—O5W	135.30 (19)	K1^vii^—O4B—K4B	95.3 (3)
O3W^v^—K3B—O5W	89.0 (2)	C2B—O4B—K4A	101.84 (12)
O7—K3B—N2^iv^	96.10 (18)	K1^vii^—O4B—K4A	90.10 (5)
O1W^iv^—K3B—N2^iv^	125.9 (2)	C4B—O5B—C5B	109.77 (15)
O7B—K3B—N2^iv^	87.4 (2)	C6B—O6B—K2^iv^	108.65 (13)
O4WA—K3B—N2^iv^	111.6 (4)	C6B—O6B—K1^iv^	119.80 (13)
O4^iv^—K3B—N2^iv^	56.26 (13)	K2^iv^—O6B—K1^iv^	95.01 (5)
O3W^v^—K3B—N2^iv^	96.2 (4)	C6B—O6B—K4B^ix^	98.8 (3)
O5W—K3B—N2^iv^	167.8 (4)	K2^iv^—O6B—K4B^ix^	150.5 (3)
O7—K3B—C2^iv^	136.7 (3)	K1^iv^—O6B—K4B^ix^	80.1 (3)
O1W^iv^—K3B—C2^iv^	85.01 (14)	C7B—O7B—K4A^ix^	114.78 (13)
O7B—K3B—C2^iv^	67.41 (13)	C7B—O7B—K3B	120.48 (16)
O4WA—K3B—C2^iv^	121.9 (4)	K4A^ix^—O7B—K3B	123.07 (13)
O4^iv^—K3B—C2^iv^	19.60 (5)	C7B—O7B—K3A	120.27 (16)
O3W^v^—K3B—C2^iv^	100.7 (4)	K4A^ix^—O7B—K3A	123.98 (10)
O5W—K3B—C2^iv^	148.1 (2)	C7B—O7B—K1^iv^	110.33 (13)
N2^iv^—K3B—C2^iv^	41.43 (8)	K4A^ix^—O7B—K1^iv^	82.14 (4)
O7—K3B—K1^iv^	172.3 (2)	K3B—O7B—K1^iv^	90.9 (2)
O1W^iv^—K3B—K1^iv^	39.15 (8)	K3A—O7B—K1^iv^	88.18 (8)
O7B—K3B—K1^iv^	48.23 (8)	C7B—O7B—K4B^ix^	104.3 (3)
O4WA—K3B—K1^iv^	102.6 (3)	K3B—O7B—K4B^ix^	134.9 (3)
O4^iv^—K3B—K1^iv^	43.58 (8)	K3A—O7B—K4B^ix^	135.4 (3)
O3W^v^—K3B—K1^iv^	108.7 (3)	K1^iv^—O7B—K4B^ix^	78.1 (3)
O5W—K3B—K1^iv^	97.18 (18)	C2B—N1B—N2B	116.70 (16)
N2^iv^—K3B—K1^iv^	91.58 (11)	C2B—N1B—Ni1B	116.37 (13)
C2^iv^—K3B—K1^iv^	50.88 (7)	N2B—N1B—Ni1B	126.63 (12)
O7—K3B—K4B^v^	89.6 (4)	N1B—N2B—C4B	110.68 (15)
O1W^iv^—K3B—K4B^v^	74.3 (4)	N1B—N2B—C3B	109.51 (14)
O7B—K3B—K4B^v^	138.6 (3)	C4B—N2B—C3B	108.53 (15)
O4WA—K3B—K4B^v^	154.1 (3)	N4B—N3B—C5B	110.58 (15)
O4^iv^—K3B—K4B^v^	68.1 (4)	N4B—N3B—C3B	109.35 (15)
O3W^v^—K3B—K4B^v^	19.4 (3)	C5B—N3B—C3B	108.84 (16)
O5W—K3B—K4B^v^	98.4 (3)	N4B—N3B—K2^iv^	104.06 (10)
N2^iv^—K3B—K4B^v^	89.9 (4)	C5B—N3B—K2^iv^	123.74 (11)
C2^iv^—K3B—K4B^v^	83.7 (4)	C3B—N3B—K2^iv^	99.25 (11)
K1^iv^—K3B—K4B^v^	90.6 (3)	C6B—N4B—N3B	116.71 (16)
O7—K3B—H1W4	87.3	C6B—N4B—Ni1B	116.48 (13)
O1W^iv^—K3B—H1W4	101.2	N3B—N4B—Ni1B	126.38 (12)
O7B—K3B—H1W4	45.0	O3B—C1B—O2B	124.92 (18)
O4WA—K3B—H1W4	16.9	O3B—C1B—C2B	119.87 (17)
O4^iv^—K3B—H1W4	119.2	O2B—C1B—C2B	115.19 (16)
O3W^v^—K3B—H1W4	154.0	O3B—C1B—K2	57.75 (10)
O5W—K3B—H1W4	71.5	O2B—C1B—K2	77.92 (10)
N2^iv^—K3B—H1W4	100.0	C2B—C1B—K2	142.67 (13)
C2^iv^—K3B—H1W4	105.0	O3B—C1B—K4A	53.12 (10)
K1^iv^—K3B—H1W4	91.2	O2B—C1B—K4A	143.73 (13)
K4B^v^—K3B—H1W4	170.0	C2B—C1B—K4A	78.17 (10)
O7—K3B—H5WB	75.7	K2—C1B—K4A	73.31 (4)
O1W^iv^—K3B—H5WB	71.6	O4B—C2B—N1B	128.98 (18)
O7B—K3B—H5WB	81.8	O4B—C2B—C1B	121.58 (17)
O4WA—K3B—H5WB	44.5	N1B—C2B—C1B	109.44 (16)
O4^iv^—K3B—H5WB	139.6	O4B—C2B—K4A	56.53 (11)
O3W^v^—K3B—H5WB	102.2	N1B—C2B—K4A	147.78 (14)
O5W—K3B—H5WB	14.0	C1B—C2B—K4A	74.78 (10)
N2^iv^—K3B—H5WB	155.9	O4B—C2B—K4B	52.6 (3)
C2^iv^—K3B—H5WB	145.6	N1B—C2B—K4B	136.4 (3)
K1^iv^—K3B—H5WB	97.1	C1B—C2B—K4B	86.4 (3)
K4B^v^—K3B—H5WB	112.4	N3B—C3B—N2B	114.29 (15)
H1W4—K3B—H5WB	57.6	N3B—C3B—H3B1	108.7
O3W—K4A—O3B	144.09 (5)	N2B—C3B—H3B1	108.7
O3W—K4A—O7B^vi^	116.45 (5)	N3B—C3B—H3B2	108.7
O3B—K4A—O7B^vi^	99.01 (5)	N2B—C3B—H3B2	108.7
O3W—K4A—O4B	106.80 (6)	H3B1—C3B—H3B2	107.6
O3B—K4A—O4B	60.94 (4)	O5B—C4B—N2B	113.06 (16)
O7B^vi^—K4A—O4B	96.81 (5)	O5B—C4B—H4B1	109.0
O3W—K4A—O2W	104.92 (6)	N2B—C4B—H4B1	109.0
O3B—K4A—O2W	78.94 (5)	O5B—C4B—H4B2	109.0
O7B^vi^—K4A—O2W	90.47 (5)	N2B—C4B—H4B2	109.0
O4B—K4A—O2W	139.85 (5)	H4B1—C4B—H4B2	107.8
O3W—K4A—O6^iv^	70.42 (5)	O5B—C5B—N3B	112.88 (16)
O3B—K4A—O6^iv^	76.37 (5)	O5B—C5B—H5B1	109.0
O7B^vi^—K4A—O6^iv^	165.73 (5)	N3B—C5B—H5B1	109.0
O4B—K4A—O6^iv^	92.69 (4)	O5B—C5B—H5B2	109.0
O2W—K4A—O6^iv^	75.44 (5)	N3B—C5B—H5B2	109.0
O3W—K4A—C1B	125.55 (5)	H5B1—C5B—H5B2	107.8
O3B—K4A—C1B	21.38 (4)	O6B—C6B—N4B	129.67 (19)
O7B^vi^—K4A—C1B	113.59 (5)	O6B—C6B—C7B	121.00 (18)
O4B—K4A—C1B	46.47 (4)	N4B—C6B—C7B	109.32 (16)
O2W—K4A—C1B	94.60 (5)	O6B—C6B—K2^iv^	51.34 (11)
O6^iv^—K4A—C1B	66.25 (5)	N4B—C6B—K2^iv^	86.86 (12)
O3W—K4A—C2B	110.36 (5)	C7B—C6B—K2^iv^	146.62 (13)
O3B—K4A—C2B	45.75 (5)	O7B—C7B—O1B	124.88 (19)
O7B^vi^—K4A—C2B	112.13 (5)	O7B—C7B—C6B	119.26 (18)
O4B—K4A—C2B	21.63 (4)	O1B—C7B—C6B	115.85 (17)
O2W—K4A—C2B	121.52 (5)	O7B—C7B—K4A^ix^	46.30 (11)
O6^iv^—K4A—C2B	74.55 (5)	O1B—C7B—K4A^ix^	132.10 (13)
C1B—K4A—C2B	27.05 (5)	C6B—C7B—K4A^ix^	91.96 (11)
			
O3B—K4A—K4B—O3W	−136.2 (12)	K2—C1—C2—N1	92.1 (2)
O7B^vi^—K4A—K4B—O3W	97.0 (17)	K1—C1—C2—N1	143.32 (14)
O4B—K4A—K4B—O3W	−172.4 (19)	O3—C1—C2—K1	38.29 (16)
O2W—K4A—K4B—O3W	−23 (2)	O2—C1—C2—K1	−140.55 (16)
O6^iv^—K4A—K4B—O3W	−75.9 (16)	K2—C1—C2—K1	−51.2 (2)
C1B—K4A—K4B—O3W	−135.8 (14)	O3—C1—C2—K3A^iii^	59.6 (3)
C2B—K4A—K4B—O3W	−151.6 (18)	O2—C1—C2—K3A^iii^	−119.2 (2)
C7B^vi^—K4A—K4B—O3W	85.3 (17)	K2—C1—C2—K3A^iii^	−29.9 (4)
K2—K4A—K4B—O3W	−80.6 (18)	K1—C1—C2—K3A^iii^	21.3 (2)
K1^vii^—K4A—K4B—O3W	146.5 (16)	O3—C1—C2—K3B^iii^	65.7 (5)
O3W—K4A—K4B—O4B	172.4 (19)	O2—C1—C2—K3B^iii^	−113.1 (4)
O3B—K4A—K4B—O4B	36.2 (10)	K2—C1—C2—K3B^iii^	−23.8 (5)
O7B^vi^—K4A—K4B—O4B	−90.7 (6)	K1—C1—C2—K3B^iii^	27.4 (4)
O2W—K4A—K4B—O4B	149.0 (7)	N4—N3—C3—N2	−69.27 (19)
O6^iv^—K4A—K4B—O4B	96.5 (4)	C5—N3—C3—N2	51.86 (19)
C1B—K4A—K4B—O4B	36.6 (5)	K2^iii^—N3—C3—N2	174.98 (11)
C2B—K4A—K4B—O4B	20.78 (14)	N1—N2—C3—N3	68.50 (19)
C7B^vi^—K4A—K4B—O4B	−102.3 (3)	C4—N2—C3—N3	−52.22 (19)
K2—K4A—K4B—O4B	91.7 (7)	K3A^iii^—N2—C3—N3	−171.85 (14)
K1^vii^—K4A—K4B—O4B	−41.1 (3)	K3B^iii^—N2—C3—N3	−175.6 (3)
O3W—K4A—K4B—O6^iv^	75.9 (16)	C5—O5—C4—N2	−59.19 (19)
O3B—K4A—K4B—O6^iv^	−60.3 (10)	N1—N2—C4—O5	−64.63 (19)
O7B^vi^—K4A—K4B—O6^iv^	172.8 (3)	C3—N2—C4—O5	55.24 (19)
O4B—K4A—K4B—O6^iv^	−96.5 (4)	K3A^iii^—N2—C4—O5	179.06 (14)
O2W—K4A—K4B—O6^iv^	52.5 (11)	K3B^iii^—N2—C4—O5	177.64 (17)
C1B—K4A—K4B—O6^iv^	−59.9 (5)	C4—O5—C5—N3	59.2 (2)
C2B—K4A—K4B—O6^iv^	−75.7 (3)	N4—N3—C5—O5	65.7 (2)
C7B^vi^—K4A—K4B—O6^iv^	161.16 (10)	C3—N3—C5—O5	−54.8 (2)
K2—K4A—K4B—O6^iv^	−4.8 (9)	K2^iii^—N3—C5—O5	−171.15 (11)
K1^vii^—K4A—K4B—O6^iv^	−137.6 (2)	K4B^iii^—O6—C6—N4	151.4 (4)
O3W—K4A—K4B—O7^iv^	103 (3)	K2^iii^—O6—C6—N4	33.0 (3)
O3B—K4A—K4B—O7^iv^	−33 (3)	K4A^iii^—O6—C6—N4	135.77 (18)
O7B^vi^—K4A—K4B—O7^iv^	−159.8 (13)	K4B^iii^—O6—C6—C7	−29.9 (5)
O4B—K4A—K4B—O7^iv^	−69.2 (17)	K2^iii^—O6—C6—C7	−148.30 (14)
O2W—K4A—K4B—O7^iv^	80 (2)	K4A^iii^—O6—C6—C7	−45.5 (3)
O6^iv^—K4A—K4B—O7^iv^	27.3 (15)	K4B^iii^—O6—C6—K2^iii^	118.4 (4)
C1B—K4A—K4B—O7^iv^	−33 (2)	K4A^iii^—O6—C6—K2^iii^	102.78 (16)
C2B—K4A—K4B—O7^iv^	−48.4 (17)	N3—N4—C6—O6	−0.5 (3)
C7B^vi^—K4A—K4B—O7^iv^	−171.5 (16)	Ni1—N4—C6—O6	−173.90 (17)
K2—K4A—K4B—O7^iv^	23 (2)	N3—N4—C6—C7	−179.34 (15)
K1^vii^—K4A—K4B—O7^iv^	−110.2 (18)	Ni1—N4—C6—C7	7.3 (2)
O3W—K4A—K4B—O4WB^iv^	−155 (3)	N3—N4—C6—K2^iii^	23.05 (15)
O3B—K4A—K4B—O4WB^iv^	68.6 (18)	Ni1—N4—C6—K2^iii^	−150.34 (10)
O7B^vi^—K4A—K4B—O4WB^iv^	−58.3 (17)	K3A—O7—C7—O1	12.2 (4)
O4B—K4A—K4B—O4WB^iv^	32.4 (12)	K3B—O7—C7—O1	4.8 (6)
O2W—K4A—K4B—O4WB^iv^	−178.6 (5)	K4B^iii^—O7—C7—O1	−151.0 (4)
O6^iv^—K4A—K4B—O4WB^iv^	128.9 (15)	K3A—O7—C7—C6	−167.15 (17)
C1B—K4A—K4B—O4WB^iv^	69.0 (15)	K3B—O7—C7—C6	−174.6 (5)
C2B—K4A—K4B—O4WB^iv^	53.2 (13)	K4B^iii^—O7—C7—C6	29.6 (4)
C7B^vi^—K4A—K4B—O4WB^iv^	−69.9 (14)	Ni1—O1—C7—O7	179.31 (18)
K2—K4A—K4B—O4WB^iv^	124.2 (12)	Ni1—O1—C7—C6	−1.3 (2)
K1^vii^—K4A—K4B—O4WB^iv^	−8.7 (14)	O6—C6—C7—O7	−3.3 (3)
O3W—K4A—K4B—O7B^vi^	−97.0 (17)	N4—C6—C7—O7	175.6 (2)
O3B—K4A—K4B—O7B^vi^	126.8 (13)	K2^iii^—C6—C7—O7	−60.2 (4)
O4B—K4A—K4B—O7B^vi^	90.7 (6)	O6—C6—C7—O1	177.25 (18)
O2W—K4A—K4B—O7B^vi^	−120.4 (13)	N4—C6—C7—O1	−3.8 (2)
O6^iv^—K4A—K4B—O7B^vi^	−172.8 (3)	K2^iii^—C6—C7—O1	120.4 (2)
C1B—K4A—K4B—O7B^vi^	127.3 (8)	N4B—Ni1B—O1B—C7B	6.27 (14)
C2B—K4A—K4B—O7B^vi^	111.5 (5)	O2B—Ni1B—O1B—C7B	−171.86 (14)
C7B^vi^—K4A—K4B—O7B^vi^	−11.7 (3)	N1B—Ni1B—O2B—C1B	9.98 (14)
K2—K4A—K4B—O7B^vi^	−177.6 (12)	O1B—Ni1B—O2B—C1B	−169.56 (14)
K1^vii^—K4A—K4B—O7B^vi^	49.6 (5)	N1B—Ni1B—O2B—K2	−99.18 (14)
O3W—K4A—K4B—O5W^iv^	−110 (3)	O1B—Ni1B—O2B—K2	81.28 (13)
O3B—K4A—K4B—O5W^iv^	113.5 (19)	N4B—Ni1B—N1B—C2B	172.86 (15)
O7B^vi^—K4A—K4B—O5W^iv^	−13 (3)	O2B—Ni1B—N1B—C2B	−9.00 (15)
O4B—K4A—K4B—O5W^iv^	77 (2)	N4B—Ni1B—N1B—N2B	−0.66 (16)
O2W—K4A—K4B—O5W^iv^	−133.8 (18)	O2B—Ni1B—N1B—N2B	177.48 (16)
O6^iv^—K4A—K4B—O5W^iv^	174 (2)	C2B—N1B—N2B—C4B	−83.8 (2)
C1B—K4A—K4B—O5W^iv^	114 (2)	Ni1B—N1B—N2B—C4B	89.67 (18)
C2B—K4A—K4B—O5W^iv^	98 (2)	C2B—N1B—N2B—C3B	156.56 (17)
C7B^vi^—K4A—K4B—O5W^iv^	−25 (2)	Ni1B—N1B—N2B—C3B	−29.9 (2)
K2—K4A—K4B—O5W^iv^	169.0 (15)	C5B—N3B—N4B—C6B	98.5 (2)
K1^vii^—K4A—K4B—O5W^iv^	36 (2)	C3B—N3B—N4B—C6B	−141.70 (17)
O3W—K4A—K4B—O3B	136.2 (12)	K2^iv^—N3B—N4B—C6B	−36.42 (18)
O7B^vi^—K4A—K4B—O3B	−126.8 (13)	C5B—N3B—N4B—Ni1B	−89.30 (18)
O4B—K4A—K4B—O3B	−36.2 (10)	C3B—N3B—N4B—Ni1B	30.5 (2)
O2W—K4A—K4B—O3B	112.8 (14)	K2^iv^—N3B—N4B—Ni1B	135.82 (11)
O6^iv^—K4A—K4B—O3B	60.3 (10)	N1B—Ni1B—N4B—C6B	172.60 (15)
C1B—K4A—K4B—O3B	0.4 (5)	O1B—Ni1B—N4B—C6B	−7.82 (15)
C2B—K4A—K4B—O3B	−15.4 (9)	N1B—Ni1B—N4B—N3B	0.35 (17)
C7B^vi^—K4A—K4B—O3B	−138.5 (10)	O1B—Ni1B—N4B—N3B	179.92 (16)
K2—K4A—K4B—O3B	55.6 (6)	K4A—O3B—C1B—O2B	134.87 (18)
K1^vii^—K4A—K4B—O3B	−77.3 (8)	K2—O3B—C1B—O2B	42.1 (2)
O3W—K4A—K4B—C2B	151.6 (18)	K4B—O3B—C1B—O2B	135.1 (3)
O3B—K4A—K4B—C2B	15.4 (9)	K4A—O3B—C1B—C2B	−43.4 (2)
O7B^vi^—K4A—K4B—C2B	−111.5 (5)	K2—O3B—C1B—C2B	−136.21 (15)
O4B—K4A—K4B—C2B	−20.78 (14)	K4B—O3B—C1B—C2B	−43.2 (3)
O2W—K4A—K4B—C2B	128.2 (9)	K4A—O3B—C1B—K2	92.80 (8)
O6^iv^—K4A—K4B—C2B	75.7 (3)	K4B—O3B—C1B—K2	93.0 (3)
C1B—K4A—K4B—C2B	15.8 (4)	K2—O3B—C1B—K4A	−92.80 (8)
C7B^vi^—K4A—K4B—C2B	−123.12 (18)	K4B—O3B—C1B—K4A	0.2 (3)
K2—K4A—K4B—C2B	71.0 (7)	Ni1B—O2B—C1B—O3B	172.58 (16)
K1^vii^—K4A—K4B—C2B	−61.87 (15)	K2—O2B—C1B—O3B	−35.41 (19)
O3W—K4A—K4B—O6B^vi^	−108.1 (18)	Ni1B—O2B—C1B—C2B	−9.1 (2)
O3B—K4A—K4B—O6B^vi^	115.7 (7)	K2—O2B—C1B—C2B	142.93 (16)
O7B^vi^—K4A—K4B—O6B^vi^	−11.2 (10)	Ni1B—O2B—C1B—K2	−152.01 (9)
O4B—K4A—K4B—O6B^vi^	79.5 (5)	Ni1B—O2B—C1B—K4A	−114.02 (18)
O2W—K4A—K4B—O6B^vi^	−131.5 (8)	K2—O2B—C1B—K4A	37.99 (18)
O6^iv^—K4A—K4B—O6B^vi^	176.0 (7)	K1^vii^—O4B—C2B—N1B	113.4 (2)
C1B—K4A—K4B—O6B^vi^	116.1 (4)	K4B—O4B—C2B—N1B	−123.6 (4)
C2B—K4A—K4B—O6B^vi^	100.3 (5)	K4A—O4B—C2B—N1B	−140.3 (2)
C7B^vi^—K4A—K4B—O6B^vi^	−22.8 (6)	K1^vii^—O4B—C2B—C1B	−67.2 (3)
K2—K4A—K4B—O6B^vi^	171.2 (3)	K4B—O4B—C2B—C1B	55.8 (4)
K1^vii^—K4A—K4B—O6B^vi^	38.4 (4)	K4A—O4B—C2B—C1B	39.1 (2)
O3W—K4A—K4B—K1^vii^	−146.5 (16)	K1^vii^—O4B—C2B—K4A	−106.3 (2)
O3B—K4A—K4B—K1^vii^	77.3 (8)	K4B—O4B—C2B—K4A	16.7 (4)
O7B^vi^—K4A—K4B—K1^vii^	−49.6 (5)	K1^vii^—O4B—C2B—K4B	−123.0 (5)
O4B—K4A—K4B—K1^vii^	41.1 (3)	K4A—O4B—C2B—K4B	−16.7 (4)
O2W—K4A—K4B—K1^vii^	−170.0 (10)	N2B—N1B—C2B—O4B	−0.2 (3)
O6^iv^—K4A—K4B—K1^vii^	137.6 (2)	Ni1B—N1B—C2B—O4B	−174.38 (18)
C1B—K4A—K4B—K1^vii^	77.7 (3)	N2B—N1B—C2B—C1B	−179.70 (15)
C2B—K4A—K4B—K1^vii^	61.87 (15)	Ni1B—N1B—C2B—C1B	6.1 (2)
C7B^vi^—K4A—K4B—K1^vii^	−61.3 (2)	N2B—N1B—C2B—K4A	−87.5 (3)
K2—K4A—K4B—K1^vii^	132.8 (7)	Ni1B—N1B—C2B—K4A	98.3 (2)
N4—Ni1—O1—C7	4.19 (14)	N2B—N1B—C2B—K4B	−73.8 (5)
O2—Ni1—O1—C7	−174.48 (14)	Ni1B—N1B—C2B—K4B	112.1 (4)
N4—Ni1—N1—C2	175.16 (14)	O3B—C1B—C2B—O4B	1.1 (3)
O2—Ni1—N1—C2	−6.16 (14)	O2B—C1B—C2B—O4B	−177.33 (18)
N4—Ni1—N1—N2	−1.06 (16)	K2—C1B—C2B—O4B	−73.7 (3)
O2—Ni1—N1—N2	177.62 (15)	K4A—C1B—C2B—O4B	−33.06 (18)
C2—N1—N2—C4	−86.08 (19)	O3B—C1B—C2B—N1B	−179.34 (18)
Ni1—N1—N2—C4	90.13 (17)	O2B—C1B—C2B—N1B	2.2 (2)
C2—N1—N2—C3	154.28 (16)	K2—C1B—C2B—N1B	105.8 (2)
Ni1—N1—N2—C3	−29.5 (2)	K4A—C1B—C2B—N1B	146.49 (15)
C2—N1—N2—K3A^iii^	30.35 (19)	O3B—C1B—C2B—K4A	34.17 (17)
Ni1—N1—N2—K3A^iii^	−153.43 (12)	O2B—C1B—C2B—K4A	−144.27 (17)
C2—N1—N2—K3B^iii^	35.4 (4)	K2—C1B—C2B—K4A	−40.66 (17)
Ni1—N1—N2—K3B^iii^	−148.4 (3)	O3B—C1B—C2B—K4B	42.3 (3)
C5—N3—N4—C6	98.02 (19)	O2B—C1B—C2B—K4B	−136.1 (3)
C3—N3—N4—C6	−142.21 (17)	K2—C1B—C2B—K4B	−32.5 (3)
K2^iii^—N3—N4—C6	−29.02 (19)	K4A—C1B—C2B—K4B	8.1 (3)
C5—N3—N4—Ni1	−89.34 (18)	N4B—N3B—C3B—N2B	−69.1 (2)
C3—N3—N4—Ni1	30.4 (2)	C5B—N3B—C3B—N2B	51.8 (2)
K2^iii^—N3—N4—Ni1	143.62 (10)	K2^iv^—N3B—C3B—N2B	−177.62 (12)
N1—Ni1—N4—C6	173.36 (15)	N1B—N2B—C3B—N3B	68.8 (2)
O1—Ni1—N4—C6	−6.73 (14)	C4B—N2B—C3B—N3B	−52.1 (2)
N1—Ni1—N4—N3	0.65 (16)	C5B—O5B—C4B—N2B	−59.4 (2)
O1—Ni1—N4—N3	−179.44 (15)	N1B—N2B—C4B—O5B	−64.8 (2)
K1^ii^—O3—C1—O2	−105.7 (2)	C3B—N2B—C4B—O5B	55.4 (2)
K2—O3—C1—O2	33.8 (2)	C4B—O5B—C5B—N3B	58.9 (2)
K1—O3—C1—O2	130.80 (17)	N4B—N3B—C5B—O5B	65.6 (2)
K1^ii^—O3—C1—C2	75.6 (3)	C3B—N3B—C5B—O5B	−54.5 (2)
K2—O3—C1—C2	−144.95 (15)	K2^iv^—N3B—C5B—O5B	−170.11 (11)
K1—O3—C1—C2	−47.91 (18)	K2^iv^—O6B—C6B—N4B	41.0 (3)
K1^ii^—O3—C1—K2	−139.4 (2)	K1^iv^—O6B—C6B—N4B	148.43 (18)
K1—O3—C1—K2	97.04 (7)	K4B^ix^—O6B—C6B—N4B	−128.0 (4)
K1^ii^—O3—C1—K1	123.5 (2)	K2^iv^—O6B—C6B—C7B	−140.07 (15)
K2—O3—C1—K1	−97.04 (7)	K1^iv^—O6B—C6B—C7B	−32.6 (2)
Ni1—O2—C1—O3	173.77 (16)	K4B^ix^—O6B—C6B—C7B	50.9 (4)
K2—O2—C1—O3	−32.75 (19)	K1^iv^—O6B—C6B—K2^iv^	107.42 (14)
Ni1—O2—C1—C2	−7.5 (2)	K4B^ix^—O6B—C6B—K2^iv^	−169.0 (3)
K2—O2—C1—C2	146.01 (14)	N3B—N4B—C6B—O6B	−0.7 (3)
Ni1—O2—C1—K2	−153.48 (10)	Ni1B—N4B—C6B—O6B	−173.75 (18)
Ni1—O2—C1—K1	−110.71 (16)	N3B—N4B—C6B—C7B	−179.74 (15)
K2—O2—C1—K1	42.77 (17)	Ni1B—N4B—C6B—C7B	7.2 (2)
K3A^iii^—O4—C2—N1	−34.1 (3)	N3B—N4B—C6B—K2^iv^	30.15 (15)
K3B^iii^—O4—C2—N1	−38.5 (4)	Ni1B—N4B—C6B—K2^iv^	−142.88 (10)
K1—O4—C2—N1	−137.41 (19)	K4A^ix^—O7B—C7B—O1B	117.66 (19)
K3A^iii^—O4—C2—C1	145.53 (17)	K3B—O7B—C7B—O1B	−48.1 (4)
K3B^iii^—O4—C2—C1	141.1 (3)	K3A—O7B—C7B—O1B	−51.5 (3)
K1—O4—C2—C1	42.23 (18)	K1^iv^—O7B—C7B—O1B	−151.74 (17)
K3A^iii^—O4—C2—K1	103.30 (14)	K4B^ix^—O7B—C7B—O1B	125.8 (4)
K3B^iii^—O4—C2—K1	98.9 (3)	K4A^ix^—O7B—C7B—C6B	−61.2 (2)
K3B^iii^—O4—C2—K3A^iii^	−4.4 (3)	K3B—O7B—C7B—C6B	133.0 (3)
K1—O4—C2—K3A^iii^	−103.30 (14)	K3A—O7B—C7B—C6B	129.56 (17)
K3A^iii^—O4—C2—K3B^iii^	4.4 (3)	K1^iv^—O7B—C7B—C6B	29.4 (2)
K1—O4—C2—K3B^iii^	−98.9 (3)	K4B^ix^—O7B—C7B—C6B	−53.1 (4)
N2—N1—C2—O4	−0.1 (3)	K3B—O7B—C7B—K4A^ix^	−165.7 (3)
Ni1—N1—C2—O4	−176.74 (17)	K3A—O7B—C7B—K4A^ix^	−169.2 (2)
N2—N1—C2—C1	−179.80 (15)	K1^iv^—O7B—C7B—K4A^ix^	90.60 (11)
Ni1—N1—C2—C1	3.6 (2)	K4B^ix^—O7B—C7B—K4A^ix^	8.2 (3)
N2—N1—C2—K1	−88.3 (2)	Ni1B—O1B—C7B—O7B	177.15 (17)
Ni1—N1—C2—K1	95.1 (2)	Ni1B—O1B—C7B—C6B	−3.9 (2)
N2—N1—C2—K3A^iii^	−24.43 (16)	Ni1B—O1B—C7B—K4A^ix^	−123.19 (13)
Ni1—N1—C2—K3A^iii^	158.96 (12)	O6B—C6B—C7B—O7B	−2.1 (3)
N2—N1—C2—K3B^iii^	−28.6 (3)	N4B—C6B—C7B—O7B	177.02 (18)
Ni1—N1—C2—K3B^iii^	154.8 (3)	K2^iv^—C6B—C7B—O7B	−67.7 (3)
O3—C1—C2—O4	1.9 (3)	O6B—C6B—C7B—O1B	178.91 (19)
O2—C1—C2—O4	−176.93 (17)	N4B—C6B—C7B—O1B	−2.0 (2)
K2—C1—C2—O4	−87.6 (3)	K2^iv^—C6B—C7B—O1B	113.3 (2)
K1—C1—C2—O4	−36.38 (17)	O6B—C6B—C7B—K4A^ix^	−41.5 (2)
O3—C1—C2—N1	−178.40 (17)	N4B—C6B—C7B—K4A^ix^	137.66 (14)
O2—C1—C2—N1	2.8 (2)	K2^iv^—C6B—C7B—K4A^ix^	−107.1 (2)

Symmetry codes: (i) *x*−1/2, −*y*+1/2, −*z*+1; (ii) −*x*+1, −*y*, −*z*+1; (iii) *x*−1/2, *y*, −*z*+1/2; (iv) *x*+1/2, *y*, −*z*+1/2; (v) −*x*+3/2, −*y*, *z*−1/2; (vi) *x*, −*y*+1/2, *z*+1/2; (vii) *x*+1/2, −*y*+1/2, −*z*+1; (viii) −*x*+3/2, −*y*, *z*+1/2; (ix) *x*, −*y*+1/2, *z*−1/2.

### Hydrogen-bond geometry (Å, º)

**Table:**

*D*—H···*A*	*D*—H	H···*A*	*D*···*A*	*D*—H···*A*
O1*W*—H1*W*1···O6^x^	0.92	1.94	2.840 (2)	166
O1*W*—H2*W*1···O5*W*^iii^	0.83	2.61	3.120 (4)	121
O1*W*—H2*W*1···O3*B*^i^	0.83	2.23	3.001 (2)	154
O2*W*—H2*W*2···O4^ii^	0.93	1.83	2.754 (2)	171
O4*WA*—H2*W*4···O4*B*^iii^	0.91	2.44	2.993 (2)	119
O4*WA*—H2*W*4···N2*B*^iii^	0.91	2.02	2.895 (3)	161
O5*W*—H5*WC*···O6*B*^xi^	0.85	1.95	2.774 (3)	162
O4*WB*—H3*W*4···O4*B*^iii^	0.85	2.09	2.848 (9)	149
O4*WB*—H4*W*4···O6*B*^xi^	0.88	2.15	3.024 (9)	174

Symmetry codes: (i) *x*−1/2, −*y*+1/2, −*z*+1; (ii) −*x*+1, −*y*, −*z*+1; (iii) *x*−1/2, *y*, −*z*+1/2; (x) −*x*+1/2, −*y*, *z*+1/2; (xi) *x*−1/2, −*y*+1/2, −*z*.
